# Identification of a neoplastic Tfh-like cellular subset in a mouse model of angioimmunoblastic T cell lymphoma

**DOI:** 10.3389/fonc.2026.1715613

**Published:** 2026-01-30

**Authors:** Saba Mohammaei, Jiyeon Lee, Antoine Bouchard, Evany Bernal Ballesteros, Nikoletta Diamantopoulos, Lifei Hou, Eileen Remold, Junhun Cho, Woong-Kyung Suh

**Affiliations:** 1 Institut de Recherches Cliniques de Montréal (IRCM), Montréal, QC, Canada; 2 Division of Experimental Medicine, McGill University, Montréal, QC, Canada; 3 Department of Pathology, Samsung Medical Center, Seoul, Republic of Korea; 4 Molecular Biology Program, University of Montréal, Montréal, QC, Canada; 5 Department of Microbiology and Immunology, McGill University, Montréal, QC, Canada; 6 Edelweiss Immune Inc, Brookline, MA, United States; 7 Department of Anesthesiology, Boston Children’s Hospital and Harvard Medical School, Boston, MA, United States

**Keywords:** AITL, angioimmunoblastic T cell lymphoma, CXCR6, EZH2, IL-18R, sanroque, T follicular helper cell, TFH

## Abstract

**Background:**

Nodal T-follicular helper cell lymphoma, angioimmunoblastic type (nTFHL-AI, or AITL), is an aggressive peripheral T cell lymphoma without effective treatments. It has been shown that genetic and epigenetic changes lead to the expansion of neoplastic CD4^+^ T cells originating from T follicular helper (Tfh) cells, which subsequently cause B cell expansion and tumor development. However, it remains unclear if the Tfh-like cell populations contain a subset that drives tumor progression and, if so, whether such a subset may have druggable targets.

**Methods:**

Through single-cell transcriptome analysis of Tfh-like cells isolated from the spontaneously arising AITL-like tumors in *Roquin^san/+^* mice, we identified and characterized a tumor-enriched Tfh cell subset highly expressing CXCR6 and IL-18 receptor, termed “Double-Expressor (DE) Tfh” cells. Using genetic or pharmacological approaches, we depleted DE Tfh cells *in vivo* and monitored tumor size (by ultrasound imaging) and the status of DE Tfh subset (by flow cytometry). We performed NanoString gene expression profiling of AITL patient samples to identify AITL cases that are relevant to *Roquin^san/+^* mouse model.

**Results:**

DE Tfh cells expressed higher levels of Ki-67 and enhancer of zeste homolog 2 (EZH2) and proliferated more rapidly compared to other Tfh cells in an IL-18 and EZH2 dependent manner. Furthermore, DE Tfh cells engrafted better than non-DE Tfh cells and could cause B cell expansion when adoptively transferred into lymphopenic recipients. On the other hand, depletion of DE Tfh cells via *Ezh2* gene deletion, inhibition of EZH2 (using FDA-approved drug, tazemetostat), or anti-CXCR6 mAb led to tumor regression. These findings may be relevant to a subset of human AITL cases since we found that ~20-30% of AITL patient samples have concomitantly elevated expression of *CXCR6*, *IL-18R1*, and *IFNG*.

**Conclusions:**

Our study identified a pathogenic Tfh-like subset essential for AITL tumor progression in a mouse model and suggests that identifying and targeting a DE Tfh-like subset in AITL patients might be an effective strategy.

## 1 Introduction

Angioimmunoblastic T cell lymphoma (AITL) is an aggressive non-Hodgkin lymphoma with a 5-year survival rate remaining at ~33% (1, 2). Patients suffer from lymphadenopathy, splenomegaly, systemic inflammation (fever and/or skin rash), and immune dysregulation (autoimmune symptoms or immunodeficiency). Immunophenotyping and gene expression profiling established that AITL is one of the three peripheral T cell lymphomas (PTCL) originating from T follicular helper (Tfh) cells (3–6). The hallmark features of AITL are effacement of the T-B border (absence of B cell follicles), arborization of endothelial venules, and extension of follicular dendritic cell mesh (6, 7). To reflect these key pathological aspects, AITL was recently renamed as Nodal T-follicular helper cell lymphoma angioimmunoblastic type (nTFHL-AI), contrasting nTFHL follicular type (nTFHL-F) or nTFHL not otherwise specified type (nTFHL-NOS) (6). So far, there is no effective treatment for AITL (1, 8). A deeper understanding of the mechanisms that drive neoplastic transformation and maintenance of the pathogenic Tfh cell populations should facilitate the development of new therapeutic options.

Tfh cells are a subset of CD4^+^ T cells that express BCL6, CXCR5, and PD-1 that promote germinal center reactions (9). Depending on the microenvironment, Tfh cells may obtain features of Th1, Th2, or Th17 lineage, thus becoming Tfh1, Tfh2, or Tfh17 cells (10, 11). In the germinal center, Tfh cells are continuously in contact with B cells. During T-B contact, Tfh cells deliver B cell-activating signals through CD40L, IL-4, and IL-21, enabling antigen-specific B cell clones to differentiate into antibody-producing plasma cells (9). Resembling intimate T-B collaboration within the germinal centers, AITL tumor Tfh-like cells appear to rely on T-B interactions for tumors to grow (12–14).

Exome sequencing of AITL tumor samples revealed several highly recurrent somatic mutations (15–17). The emerging mutational landscape of AITL supports a multi-step model (1, 16, 18). Firstly, *TET2* or *DNMT3A* genes are mutated in hematopoietic progenitor cells, and these mutations predispose myeloid and lymphoid precursor cells to neoplastic transformation. Second, subsequent driver mutations such as *RHOA G17V* (up to 70% of AITL cases) or *IDH2* in mature peripheral CD4^+^ T cells may allow oligoclonal expansion of Tfh cells. Lastly, inflammation caused by Tfh and other immune and non-immune cells appears to foster AITL tumor growth (1). Although rare, some AITL cases can arise without mutations in *TET2*, *DNMT3A, RHOA, or IDH2* but have mutations in genes encoding PLCγ1, CD28, FYN, and PI3K elements, activating TCR signaling (17, 19). Animal modeling studies indicate that these genetic and epigenetic changes cooperate to induce hyperactivation of CD4^+^ T cells, increased Tfh specification, and ultimately Tfh-derived neoplasm (12, 13, 20).

It has been shown that heterozygosity in the *Roquin1* point mutation (*sanroque* allele) leads to spontaneous formation of AITL-like tumors in mice upon aging (~40% of male and ~60% of female mice at 6 months of age) (21). We have shown that BCL6-driven Tfh signatures and close T-B crosstalk are critical for the growth of AITL-like tumors in *Roquin^san/+^* mice (14). In contrast, *sanroque* homozygosity leads to augmented expression of IFN-γ by Tfh cells, which leads to expansion of Tfh cells, autoantibody generation, and lupus-like symptoms admixed with AITL-like tumors (22). Thus, it seems possible that tumors arising in *Roquin^san/+^* mice may have polarization of Tfh cells towards Tfh1-like cells and signs of IFN-γ-driven inflammation.

The histone methyl transferase enhancer of zeste homolog 2 (EZH2) is overexpressed in many PTCLs, including AITL, and the EZH2 expression level is positively correlated with proliferation index Ki-67 and poor prognosis in T cell neoplasms (23, 24). Additionally, EZH2 normally promotes Tfh differentiation and survival during germinal center reactions (25). Thus, EZH2 may play important roles in the progression of Tfh-derived T cell lymphomas.

Here, we found that the AITL-like tumors in *Roquin^san/+^* mice contain a group of Tfh-like cells that express high levels of CXCR6 and IL-18 receptor [termed “Double-Expressor (DE) Tfh cells”] along with an IFN-γ gene signature. Compared to other Tfh-like cells, DE Tfh cells had a superior capacity to proliferate and an ability to cause the expansion of B cells, suggesting their tumor-driving roles in AITL. These neoplastic features of DE Tfh cells relied on EZH2 function as genetic or pharmacological inactivation of EZH2 reduced the DE Tfh cell content and tumor burden. Our mouse work may have clinical implications for AITL patients since 20-30% of human AITL cases have gene signatures similar to DE Tfh cells.

## 2 Methods

### 2.1 Mice and human samples

Composite mouse lines were created by breeding mice possessing the *sanroque* allele (*Roquin^san/+^* mice) (21) supplied by C. Vinuesa from the Australian National University, with different strains of mice. CD4-CreERT2 mice (26) were used to perform tamoxifen-inducible deletion of genes in a CD4^+^ T cell-specific manner. TCRβ knockout mice (Jax002118), Rag1 knockout mice (Jax002216), and EZH2 conditional knockout mice (Jax022616) were purchased from The Jackson Laboratory. Before creating the composite lines, all mice underwent backcrossing onto the C57BL/6J background for over 10 generations. We used both male and female *Roquin^san/+^* mice for this study. Except for a lower tumor incidence in male vs female *Roquin^san/+^* mice (~40% vs ~60%, respectively) (21), tumors from male and female *Roquin^san/+^* mice responded equally to the experimental procedures. Mice were housed in a specific-pathogen-free environment at the animal facility of the Institut de Recherches Cliniques de Montréal (IRCM). Mice were housed in ventilated cages with standard space (100 cm^2^ per mouse) and bedding material. Food and water were supplied *ad libitum*. Soiled bedding was replaced once a week. Experimenal mice were monitored daily by animal technicians for pain. Mice in pain were examined by the IRCM veterinarian, and according to the professional assesment, they were either treated by analgesia or euthenized. For euthanasia, mice were placed in a CO_2_ chamber, followed by cervical dislocation to confirm death. Animal use protocols were reviewed and approved by the IRCM Animal Care Committee. Human samples were obtained from AITL patients of Samsung Medical Center, Seoul, Republic of Korea. The Institutional Review Board of Samsung Medical Center approved all the protocols of this study (IRB file number: 2021-01-093).

### 2.2 Antibodies, cytokines, and chemicals

Antibodies against B220 (RA3-6B2), CD279 (PD-1, J43 or 29F.1A12), FOXP3 (FJK-16s), GL7, and Ki-67 (16A8) were purchased from Biolegend. Antibodies against CD4 (GK1.5), CD95 (Jo2), EZH2 (11/EZH2), and Active Caspase-3 were from BD Biosciences. IL-18 blocking antibody (YIGIF74-1G7) was purchased from BioXcell. Anti-CXCR6 antibody was originally a rabbit-anti-mouse mAb, the Fab portion of which was grafted into mouse IgG2a Fc framework, giving rise to anti-CXCR6 mAb clone 19A5 (Edelweiss Immune Inc.). All other antibodies and streptavidin conjugate were purchased from ThermoFisher: CXCR5 (SPRCL5), CXCR6 (DANID2), CD16/CD32, FOXP3 (FJK-16s), GL7, IL-18Rα (P3TUNYA), Streptavidin-eFluor™ 450, and TCRβ. Recombinant IL-2 was purchased from Peprotech, and IL-18 was from Biolegend. Tazemetostat (EPZ-6438, 10 mM in DMSO) was purchased from MedchemExpress; tamoxifen was from Millipore Sigma; 4-OH-tamoxifen was from Sigma Aldrich; and CFSE was from ThermoFisher.

### 2.3 T and B cell isolation

To isolate CD4^+^ T cells or B cells from tumors, EasySep™ Mouse CD4^+^ T Cell Isolation Kit or EasySep™ Mouse B Cell Isolation Kit (STEMCELL Technologies) were used according to the manufacturer’s instructions.

### 2.4 Flow cytometry and sorting

Single-cell suspensions were made from tumors, lymph nodes, or spleens by mechanical disruption through a 70-μm nylon mesh filter (BD Biosciences) in PBS. Cell suspensions were stained with viability dye, and then washed and resuspended in PBS supplemented with 1% bovine serum albumin for staining. Next, cells were blocked with Fc-block antibody (anti-CD16/CD32) and then stained with primary antibodies followed by streptavidin conjugates. For intracellular staining, cells were fixed and permeabilized using fix/perm solution and buffer based on the manufacturer’s instructions (ThermoFisher). LSR Fortessa (BD Biosciences) and FlowJo (BD Biosciences) were used for sample acquisition and analysis, respectively. Sorting was performed by BD FACSAria (BD Biosciences).

### 2.5 *In vitro* assays

For proliferation assays, cells were resuspended in PBS (10–20 million/ml) and CFSE was added to the suspension followed by a 5 min incubation at 37°C. Next, cells were resuspended in RPMI complete media supplemented with IL-2 (1 ng/ml) and IL-18 (10 ng/ml). After adding DMSO or Tazemetostat, cells were cultured in the CO_2_ incubator at 37°C for up to 3 days. Cells were analyzed at 1 day of culture to get a baseline CFSE pattern; at day 3 to assess the level of CFSE dilution. Cells were acquired using LSR Fortessa and analyzed by ModFit LT™. For cell death assays, cells were cultured for 3 days and the level of activated caspase 3 was measured by flow cytometry. For IFN-γ stimulation, CD4^+^ T cells were isolated and incubated with 50µg/ml of PMA and 1µg/ml ionomycin, with 1µl/ml of Golgi plug in the CO_2_ incubator at 37°C for 4 hours.

### 2.6 Adoptive transfer experiments

A single-cell suspension of *Roquin^san/+^* tumors was enriched for CD4^+^ T cells using EasySep™ Mouse CD4+ T Cell Isolation Kit (STEMCELL Technologies) and stained for sorting. DE Tfh cells (CD4^+^CXCR5^+^PD-1^+^CXCR6^+^ IL18Rα^+^) and control Tfh cells (CD4^+^CXCR5^+^PD-1^+^CXCR6^-^ IL18Rα^-^) were sorted over 98% purity using a FACS Aria (BD Bioscience). Equal number of cells (~500, 000) were adoptively transferred into TCRβ knockout mice via tail vein injection. Eight to eighteen days later, the recipient’s spleen was taken for analysis. In some experiments, ~500, 000 DE Tfh cells or control Tfh cells were mixed with two million B cells isolated from the same tumor (by EasySep™ Mouse B cell Isolation Kit) and adoptively transferred into Rag1 knockout mice, and T/B cell engraftment was assessed seven days later.

### 2.7 *In vivo* experiments

For genetic ablation experiments, tumor-bearing mice with floxed *Ezh2* gene (*Roquin^san/+^;Cd4-cre ERT2 ^+/-^;Ezh2 ^f/f^*) were fed tamoxifen (Millipore Sigma) by oral gavage for 5 consecutive days (200 μg/g of body weight per day in corn oil). Tumor size was measured before and after tamoxifen treatment using sonographic imaging (VEVO 770; Visual Sonics). Mice were anesthetized with 5% isoflurane in oxygen using an induction chamber. Once the righting reflex is lost and breathing became deeper, the mouse was layed on a warm pad with 2% isoflurane being supplied via a nose cone throughout the imaging procedure. The images were taken by RMV 707B scan head and analyzed by VEVO software. Tumor area was calculated based on cross-sectional surface area using at least two ultrasound images at each time point. For *in vivo* EZH2 inhibition experiments, tumor-bearing *Roquin^san/+^* mice were treated with tazemetostat (prepared in 5% DMSO/0.5% CMC-Na solution; 400 mg/kg of body weight) by daily gavage for 15 consecutive days. For some mice, the oral gavage was performed for two cycles of 15 consecutive days with a 6-day rest between the cycles. For IL-18 blockade experiments, tumor-bearing *Roquin^san/+^* mice received intraperitoneal injections of IL-18 blocking antibody (200 µg/mouse) five times with two-day intervals. For the depletion of CXCR6^+^ DE Tfh cells, tumor-bearing *Roquin^san/+^* mice received anti-CXCR6 mAb 19A5 (300 µg/mouse) intraperitoneally 12 times with two-day intervals.

### 2.8 Single-cell RNA sequencing

Tumor or tumor-free lymph node (control) were isolated from *Roquin^san/+^* mice and stained to sort live CD4^+^CXCR5^+^PD-1^+^ T follicular helper-like cells (>95% purity). Sorted conventional and Tfh-like cells were mixed in a 1:10 ratio to have an internal control. A total of 13, 500 cells from *Roquin^san/+^* mice were sent for library preparation using 10×Genomics platform: Chromium Next GEM Single Cell 39 GEM, Chromium Next GEM Chip G Single Cell Kit, Library & Gel Bead Kit v3.1, and Chromium i7 Multiplex Kit. Sequencing was conducted at Genome Quebec Core Facility using an Illumina NovaSeq 6000, utilizing a flow cell S1 PE28*91.

### 2.9 Single-cell expression matrix analysis

The expression matrices were stored within an R Seurat object, accessible through the Seurat package, version 3.0 (27), simplifying the analysis process. During the filtering stage, we merged control and tumor samples and removed cells with over 10% mitochondrial RNA contamination and those expressing fewer than 200 unique genes. After log-normalizing and scaling the expression matrix, the most differentially expressed genes within the samples were identified. Then, a principal component analysis (PCA) method, focusing on the 2, 000 most variable features, was employed to reduce dimensions. From the PCA results, we selected the top 30 eigenvectors. These were utilized in building a Shared Nearest Neighbor graph. To identify clusters within this graph, we utilized the Modularity Optimizer, version 1.3.0 (28). The data from cells was projected onto a two-dimensional space using the Uniform Manifold Approximation and Projection (UMAP) method (29). From the total cell clusters, the ones without Foxp3 expression were isolated and moved into a new object to focus on T follicular helper cell clusters. The dataset is deposited in GSE303738.

### 2.10 NanoString nCounter assay

Patients diagnosed with AITL at Samsung Medical Center, Seoul, Republic of Korea, between January 2008 and December 2018 were enrolled in the study. From these, we randomly selected 48 cases for whom formalin-fixed paraffin-embedded (FFPE) blocks were available. For the NanoString nCounter assay, we used the nCounter^®^ Human Immunology V2 Panel with 579 human immune signature genes and 15 housekeeping genes (NanoString Technologies; Seattle, WA, USA). Total RNA was extracted from three to four FFPE tissue sections of 4-μm thickness from representative blocks using the High Pure RNA Paraffin kit (Roche Diagnostics, Mannheim, Germany). RNAs (200 ng) were hybridized to the target sequence-specific capture probes and fluorescence-labeled reporter probes. The mRNA-probe complexes were washed, immobilized, and quantified using fluorescence imaging. We performed two-step normalization for the gene expression matrix to remove the batch effect of nCounter gene expression. First, we performed within-normalization using the NanoStringNorm R package (options: CodeCount+Sum, Background=mean, and SampleContent=total.sum) and adjusted the outliers to the median value using the outlier R package (30). Next, gene expression matrices spanning two batches were rescaled by between-normalization using the edgeR R package, and log10 transformed expression was considered the final gene expression matrix (31). For heatmap generation and hierarchical clustering, we used software from Broad Institute website (https://software.broadinstitute.org/morpheus/).

### 2.11 Public AITL dataset analysis

Publicly available peripheral T cell lymphoma gene expression datasets were downloaded from refine.bio website (https://www.refine.bio/). We chose AITL data from GSE51521 since it showed reliable Z-scores for *IFNG (*32). We assessed the relative levels of *IFNG*, *CXCR6*, and other Th1-associated genes using Python 3.10.8.

### 2.12 Statistical analysis

Data were analyzed using GraphPad Prism 9.0.1. Unpaired Student’s *t-*test with Welch’s correction was used to compare the 2 groups. One-way ANOVA was used to compare 3 groups. Two-way ANOVA was used to analyze the time courses of tumor regression. P < 0.05 was considered statistically significant. For Simple linear regression analysis, R^2^ > 0.7 was considered a strong correlation. For the analysis of overall survival of AITL patient subsets, log-rank test was used.

## 3 Results

### 3.1 Identification of a highly proliferative Tfh-like cellular subset in *Roquin^san/+^* tumors

**Figure 1:**
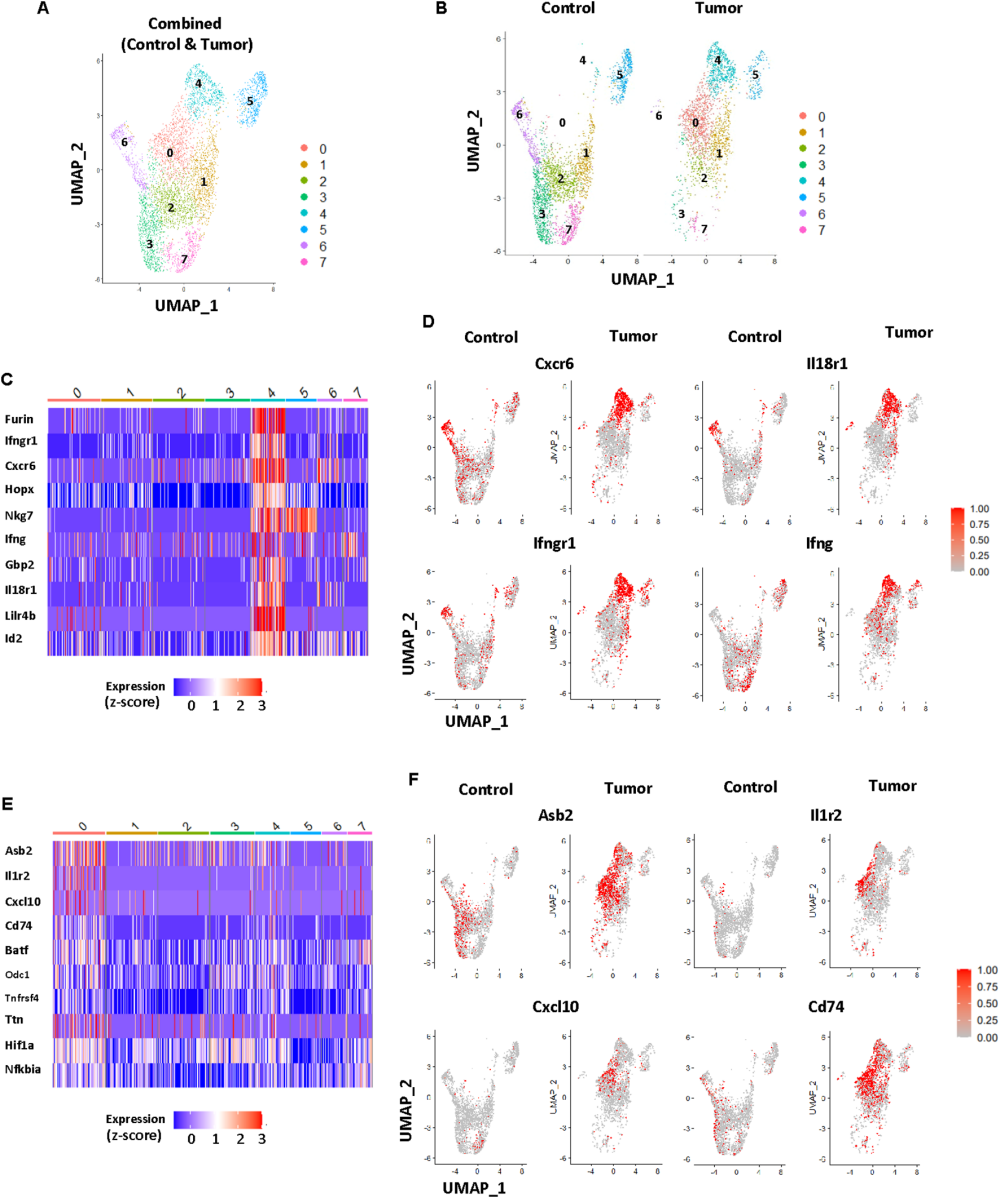
Identification of tumor-specific Tfh subsets. **(A)** UMAP projection of CD4^+^CXCR5^+^PD-1^+^Foxp3^-^ Tfh cells pooled from *Roquin^san/+^* contorl (tumor-free) and tumor samples. **(B)** UMAP projections of CD4^+^CXCR5^+^PD-1^+^Foxp3^-^ Tfh cells from *Roquin^san/+^* mice. Control, LNs from a tumor-free *Roquin^san/+^* mouse; Tumor, a tumor from a *Roquin^san/+^* mouse. Cluster 0 and Cluster 4 were most abundant in the tumor sample. **(C)** Heatmap analysis of top 10 differentially expressed genes in Cluster 4. **(D)** Feature plots of genes in Cluster 4, each dot represents a single cell. **(E)** Heatmap analysis of top ten differentially expressed genes in cluster 0. **(F)** Feature plots of genes in Cluster 0, each dot represents a single cell. For heatmaps, the gene expression values were scaled using the Seurat ScaleData function to produce Z-scores and were visualized in a heatmap using the DoHeatmap function. For feature plots, the gene expression values were log-normalized using the Seurat NormalizeData function and visualized using the FeaturePlot function.

AITL is caused by the expansion of oligoclonal neoplastic Tfh-like cells in humans and mice (1, 12, 13, 20, 21). In *Roquin^san/+^* mice, hyperactive Tfh-like cells exist even in the tumor-free stage (21). We hypothesized that tumor formation and progression are driven by distinct tumor-specific Tfh-like cell subset(s) that are highly proliferative and capable of expanding B cells. To test this idea, we FACS-sorted CD4^+^CXCR5^+^PD-1^+^ T follicular cells (Tfh and Tfr cells) from lymph nodes of tumor-free or tumor-bearing mice and performed single-cell transcriptome analysis (**Supplementary Figure 1**, **Figure 1**).

The initial UMAP projection gave 15 clusters (**Supplementary Figure 1B**). After eliminating Foxp3^+^ T follicular regulatory cell-like populations and small clusters without clear features of Tfh cells, we combined the remaining Tfh-like clusters and performed in-depth analyses (**Supplementary Figure 1C**). As shown in **Figure 1**, Tfh-like cells were grouped into eight clusters (**Figure 1A**). Among them, Cluster 0 and Cluster 4 were present in the tumor but not in tumor-free lymph nodes (**Figure 1B**). Interestingly, Cluster 4 signature genes (top ten differentially expressed genes) were highly concentrated in Cluster 4 (**Figures 1C, D**), whereas Cluster 0 signature genes were diffused across other clusters (**Figures 1E, F**). Consistent with the elevated levels of IFN-γ gene signatures in *Roquin^san/san^* mice, Tfh-like cells in Cluster 4 of *Roquin^san/+^* tumor had high levels of *Ifng*, *Ifngr1* (IFN-γ receptor), *Cxcr6*, and *Il18r1* (**Figures 1C, D**). This suggests that the *Roquin^san/+^* mouse model may represent subsets of AITL with a strong Th1 signature. In contrast, Cluster 0 signature genes did not show any strong relevance to Tfh biology or T cell lymphoma (**Figures 1E, F**).

To test whether inflammatory Tfh1-like cellular subsets exist in our mouse model, we looked at the IFN-γ production by Tfh cells (**Supplementary Figure 2**). Similar to the sanroque homozygous mouse model, Tfh cells in the *Roquin^san/+^* tumor lymph nodes showed increased IFN-γ production upon stimulation compared to Tfh cells isolated from tumor-free mice (**Supplementary Figures 2A, C**). Interestingly, ~60% of the IFN-γ^+^ Tfh cells were also positive for IL18Rα (**Supplementary Figures 2B, D**), suggesting that IL-18 signaling could play a role upstream of IFN-γ induction in these cells.

**Figure 2:**
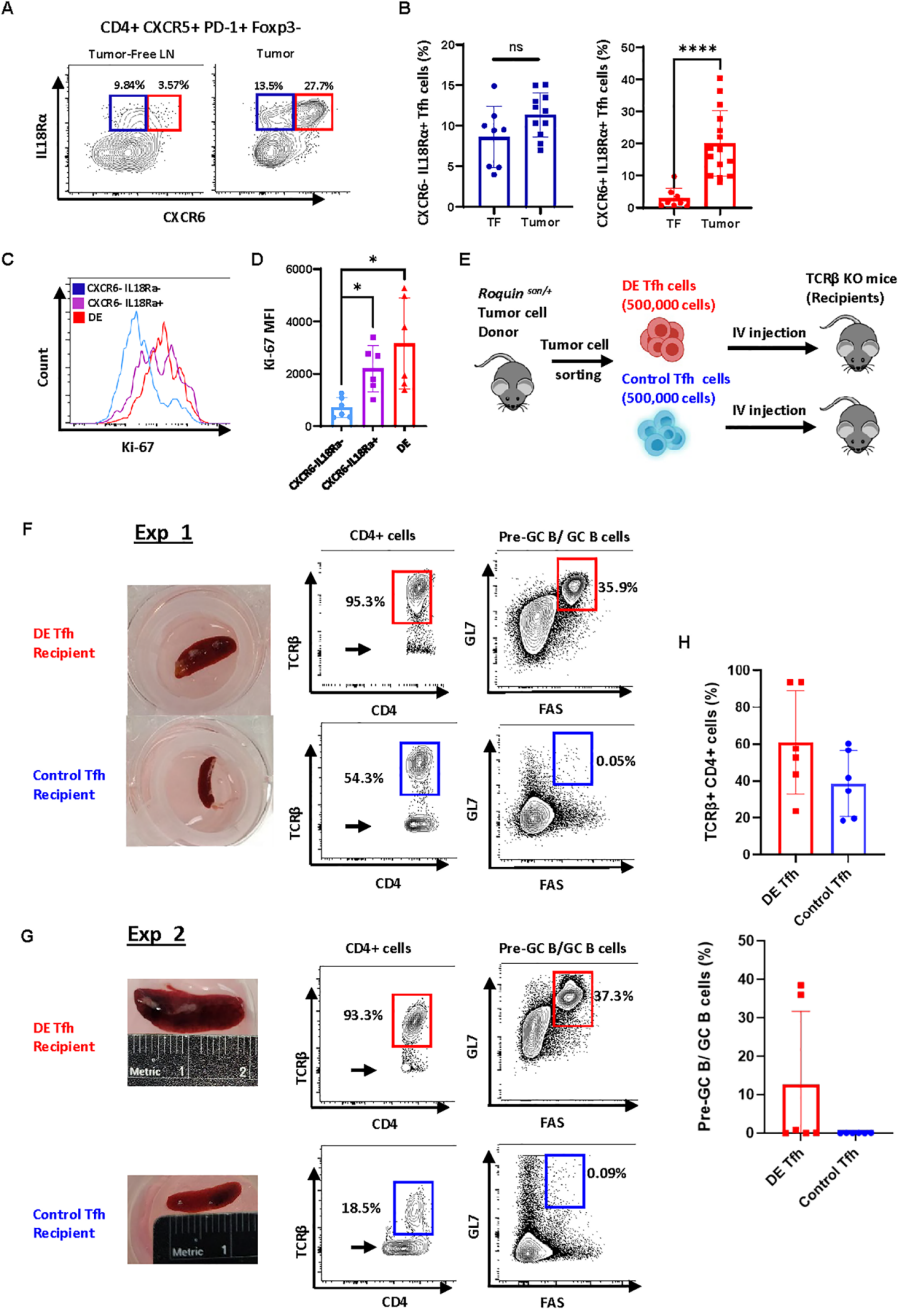
Tumor-driving capacity of CXCR6^+^IL-18R^+^ Tfh-like subset. **(A)** Cells in Cluster 4 can be identified as a CXCR6^+^IL18rα^+^ population by flow cytometry. Representative FACS plots of CXCR6 and IL18rα on live CD4^+^CXCR5^+^PD-1^+^ Foxp3^-^ cells isolated from *Roquin^san/+^* tumor-free lymph nodes or tumors are shown. **(B)** Frequencies of CXCR6^-^IL18rα^+^ and CXCR6^+^IL18rα^+^ (DE) Tfh cells in tumor-free LNs vs tumors. **(C)** Representative Ki-67 FACS plots in the indicated subsets; CXCR6^-^IL18rα^-^ (blue), CXCR6^-^IL18rα^+^ (purple), and CXCR6^+^IL18rα^+^ (DE Tfh, red). **(D)** Comparison of Ki-67 mean fluorescent intensity between CXCR6^-^IL18rα^-^ (blue), CXCR6^-^IL18rα^+^ (purple), and CXCR6^+^IL18rα^+^ (DE, red). **(E)** Experimental scheme for tumor Tfh transplantation. Single-cell suspensions of tumors from *Roquin^san/+^* mice were enriched for CD4^+^ cells and subsequently sorted by FACS (>98% purity) into DE Tfh vs control Tfh populations. Equal numbers (500, 000 each) of DE and control Tfh cells were injected into TCRβ knockout recipients (one recipient per condition). After 8 to 18 days, the spleens were taken from the recipients and analyzed. **(F, G)** Comparison of pathogenic capacities of DE vs control Tfh cells. Recipients of DE Tfh cells had splenomegaly, more vigorous expansion of transplanted TCRβ^+^ T cells (CD4^+^ T cells), and concomitant increase of an activated B cell population with FAS^+^GL7^+^ Pre-GC/GC B cell phenotype compared to control Tfh recipients in two successful experiments (**F**, Exp 1 and **G**, Exp 2) out of six such attempts. The arrows in CD4^+^ gates represent endogenous γδT cells and myeloid cells in the TCRβ knockout recipients. **(H)** Summary of TCRβ^+^CD4^+^ donor T cell contents (Top) and Pre-GC/GC B cell contents (Bottom) in DE vs Control Tfh recipeints. Data are shown as the mean ± SD, *P < 0.05, and **** < 0.0001 [**(B, H)** Student’s *t-*test; **(D)**, One-way ANOVA].

Next, we tested whether combinations of antibodies can identify cells belonging to Cluster 4 or Cluster 0 via flow cytometry. We found that cells expressing high levels of CXCR6 and IL-18 receptor [“Double-Expressor (DE) Tfh” cells hereafter] can be reliably detected and that their numbers were highly increased in tumors compared to tumor-free lymph nodes (**Figures 2A, B**). Consistent with the diffused expression pattern of the Cluster 0 signature genes, we could not find any surface markers that uniquely delineate the cells in Cluster 0 via flow cytometry.

IL-18 receptor signaling has been shown to augment the proliferation of NK cells and T cells (33). Consistently, we found that Tfh-like tumor cells expressing high levels of IL-18R had higher levels of Ki-67 compared to IL-18R-negative Tfh-like cells in the tumors (**Figures 2C, D**). To test whether DE Tfh cells have the capacity to expand B cells, we set up adoptive transfer experiments. It has been reported to be difficult to expand total CD4^+^ T cells isolated from *Roquin^san/+^* tumors after adoptive transfer (21). This is presumably due to a paucity of neoplastic cells, competition with the recipient’s T cell pool, or weak T-B collaboration in the recipients. To overcome the first two hurdles, we FACS-sorted DE Tfh cells (CD4^+^CXCR5^+^PD-1^+^CXCR6^+^IL-18Rα^+^) vs control Tfh cells (CD4^+^CXCR5^+^PD-1^+^CXCR6^-^IL-18R^-^) from tumors and adoptively transferred into T cell-deficient TCRβ knockout mice (**Figure 2E**). Even under these conditions, the engraftment succeeded only in two out of six experiments. However, in the successful experiments, only DE Tfh cells caused splenomegaly, but the control Tfh counterpart did not (**Figures 2F, G**, spleen photographs). Furthermore, DE Tfh cells expanded robustly, occupying most (>90%) of the CD4^+^ cell pool in TCRβ-deficient recipients that have only a small CD4^+^ population (presumably γδT cells and myeloid cells) (**Figures 2F, G**, CD4^+^ cells). Importantly, DE Tfh cells caused a great expansion of Fas^+^GL7^+^ B cells compared to control Tfh cells, presumably due to stronger T-B collaboration, a hallmark of AITL (**Figures 2F, G**, Pre-GC/GC B cells). In four other experiments, the contrast between DE vs control Tfh recipients was not evident in terms of splenomegaly, Tfh cell contents, and Pre-GC/GC B cell contents (**Figure 2H**).

Next, we reasoned that co-transferring DE Tfh cells along with cognate B cells may give more robust T and B cell expansion by allowing cognate T-B collaboration to continue in the recipients. To test this idea, we re-combined sorted DE Tfh cells or control Tfh cells with autologous B220^+^ B cells isolated from the same tumors (1:8 ratio) and then adoptively transferred into Rag1 knockout recipients that lack both T cell and B cell compartments (**Supplementary Figure 3**). Indeed, T cells and B cells engrafted very well in all three independent experiments. Consistent with the highly proliferative nature, DE Tfh cells showed 2-fold higher expansion in the absolute numbers over control Tfh cells (**Supplementary Figure 3A**). More than ~28% of DE Tfh cells retained the CXCR6^+^IL-18Rα^+^ phenotype, whereas control Tfh cells did not show any increase in DE Tfh markers (**Supplementary Figure 3B**). However, we could not distinguish the relative B cell helper functions of DE Tfh cells vs control Tfh cells in this model (**Supplementary Figure 3C**). This was mainly because ~25% of tumor-derived B cells showed Fas^+^GL7^+^ Pre-GC/GC B cell-like features even without co-transferred Tfh cells, and the addition of Tfh cells had only marginal impacts. We speculate that the helper functions of DE versus control Tfh cells could be more accurately evaluated by using sorted naïve B cells and by acutely immunizing Rag1 knockout recipients immediately after adoptive transfer to initiate a germinal center reaction. Nevertheless, our data showed that DE Tfh cells have better capacity to expand compared to control Tfh cells in recipients where tumor-derived B cells simultaneously expand.

Taken together, DE Tfh cells (Tfh-like cells with CXCR6^+^IL-18R^+^ phenotype in *Roquin^san/+^* tumors) closely represent the cells in Cluster 4 in the single-cell transcriptome. Based on their tumor-selective presence and highly proliferative nature *in vitro* and *in vivo*, we propose that DE Tfh cells represent a neoplastic cellular subset that is essential for tumor progression.

### 3.2 DE Tfh cells expressing EZH2 are required for tumor growth

Given that DE Tfh cells express high levels of IL-18 receptor and Ki-67, we predicted that DE Tfh cells may proliferate in response to IL-18 *in vitro*. Indeed, a combination of IL-2 and IL-18 was required to induce tumor CD4^+^ T cell proliferation *in vitro* (**Supplementary Figures 4A, B**) and the major population that responded to IL-2 plus IL-18 was DE Tfh cells (**Supplementary Figures 4C, D**). Based on IL-18-dependency of DE Tfh cells and the potential role of DE Tfh cells in promoting tumor growth, we wondered if IL-18 blockade may reduce tumor size. To test this idea, we repeatedly injected anti-IL-18 monoclonal antibodies that were known to reduce innate lymphoid cell differentiation in the bone marrow (34). However, we could not detect any signs of tumor regression with this treatment (**Supplementary Figures 4E, F**).

Next, we also tried to deplete CXCR6^+^ cells using anti-CXCR6 (clone 19A5, specific for murine CXCR6), which has been shown to deplete CXCR6-expressing T cells in mice (35) (**Supplementary Figure 5**). Indeed, about 50% of tumors (3/6) regressed after repeated injection of anti-CXCR6 mAb but the other tumors persisted (**Supplementary Figure 5A**). Intriguingly, all the regressed tumors were depleted of DE Tfh cells, whereas all the persisting tumors still maintained high levels of DE Tfh polulation (**Supplementary Figures 5B, C**). This robust correlation between the DE Tfh numbers and tumor persistence strongly supports the view that DE Tfh cells are the main driving force of tumor progression. Although it is not clear why only ~50 percent of the tumors respond to the mAb treatment, one possibility is that the delivery of antibodies into the tumors could be highly affected by disorganized tumoral vascular or lymphatic systems. Thus, we decided to search for more readily druggable targets via small molecule inhibitors.

**Figure 3:**
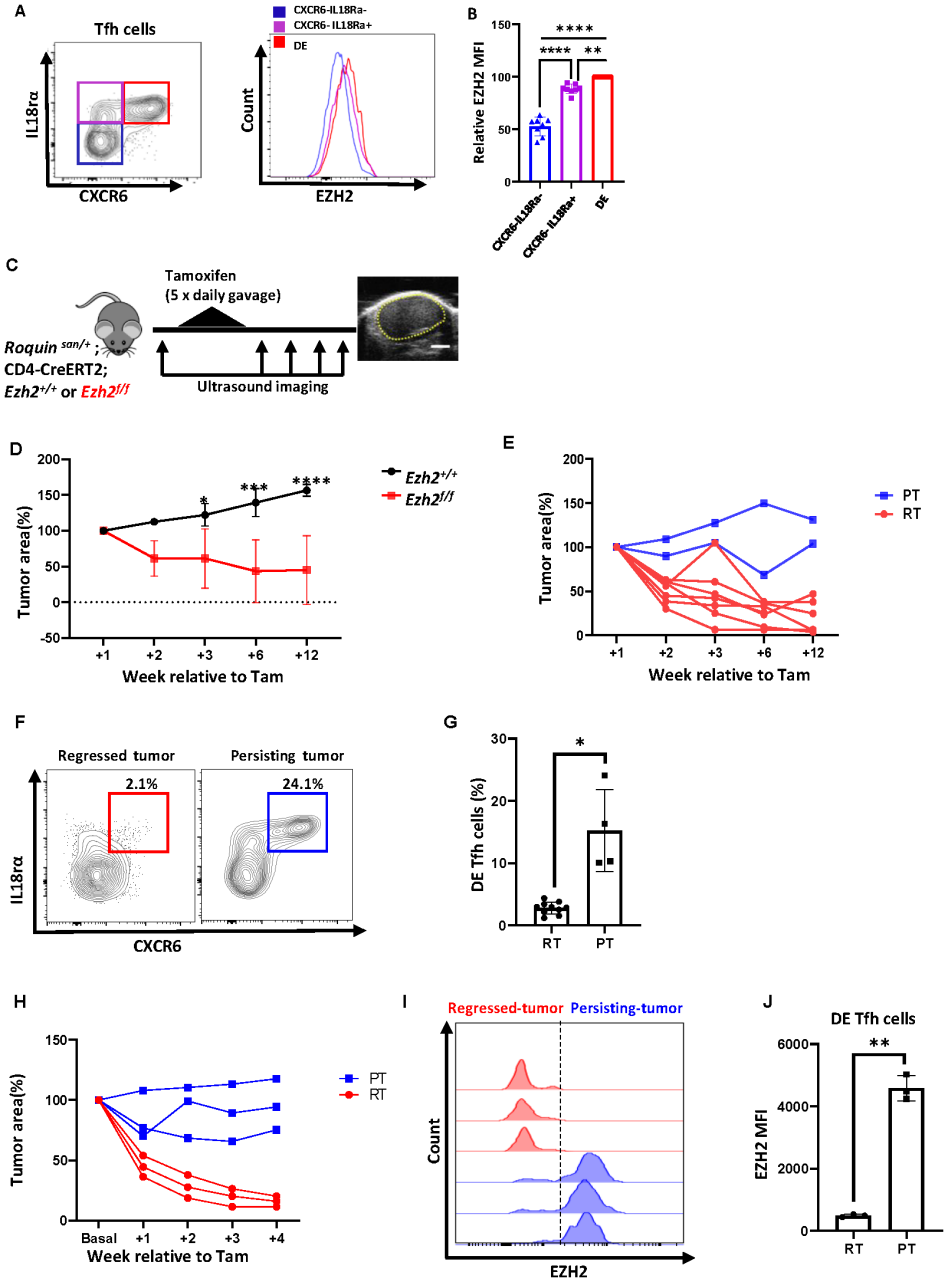
DE Tfh cells expressing EZH2 are required for tumor growth. **(A)** Elevated levels of EZH2 in DE Tfh cells compared to control Tfh cells by flow cytometric analysis. **(B)** Relative mean fluorescent intensity of EZH2 in CXCR6^-^IL18rα^-^ (blue), CXCR6^-^IL18rα^+^ (purple), and CXCR6^+^IL18rα^+^ (DE, red) Tfh compartments. **(C)** Experimental schematic of CD4-specific *Ezh2* gene deletion. *Roquin^san/+^* mice were bred with a CD4-specific Cre line to delete *Ezh2* (*Roquin^san/+^; Cd4-cre ERT2^+/-^; Ezh2^f/f^*). Ezh2 gene was acutely deleted in CD4^+^ T cells by tamoxifen treatment (5 consecutive days), and the tumor growth was monitored for up to 12 weeks using ultrasound imaging. A representative tumor sonogram is displayed. **(D)** Average time course of tumor regression in mice with *Ezh2* deletion (*Ezh2^f/f^*, n=9) compared to control (*Ezh2^+/+^*, n=3) mice. **(E)** Average time course of tumor regression in Ezh2^f/f^ mice, showing persisting tumors (PT, n=2) and regressed tumors (RT, n=7) after *Ezh2* deletion. **(F)** DE Tfh cell profiling in regressed tumors (RT) vs persisting tumors (PT) by flow cytometry and **(G)** the frequencies. **(H)** Time courses of individual tumor progression in *Ezh2^f^*^/f^ mice, showing persisting tumors (PT, n=3) and regressed tumors (RT, n=3) after *Ezh2* deletion by 3 daily tamoxifen gavage. **(I)** Histograms of EZH2 expression in DE Tfh cells from PT and RT LNs, and **(J)** a summary of EZH2 MFI. Data are shown as the mean ± SD, *P < 0.05, **P < 0.01, and ***P < 0.001, ****P < 0.0001 [**(B)** One-way ANOVA; D, Two-way ANOVA; **(G, J)** Student’s *t-*test].

Given that EZH2 is overexpressed in AITL and other peripheral T cell lymphomas (23), and that EZH2 is critical for Tfh differentiation (25), we became interested in the role of EZH2 in *Roquin^san/+^* AITL-like disease. Moreover, there is an FDA-approved EZH2 inhibitor, tazemetostat, for the treatment of B-cell lymphomas (36), which could be easily repurposed to treat other cancers. Indeed, we found that DE Tfh cells express elevated levels of EZH2 compared to other Tfh compartments (**Figures 3A, B**), suggesting their functional reliance on EZH2. However, EZH2 mRNA levels were comparable across all clusters in our scRNA-seq dataset, with no evident increase in Cluster 4. In addition, we could not detect any increase in the global H3K27me3 level in DE Tfh cells by flow cytometric analysis. These observations suggest that EZH2 expression in DE Tfh cells may be regulated post-transcriptionally, resulting in subtle epigenetic changes rather than major alterations in overall H3K27me3 content. To test if acute deletion of *Ezh2* gene in the CD4^+^ T cells in the middle of tumor growth may regress the tumors, we created a mouse model in which tamoxifen-mediated activation of Cre removes the floxed *Ezh2* gene selectively in the CD4^+^ T cell compartment (*Roquin^san/+^;Cd4-cre ERT2 ^+/-^;Ezh2 ^f/f^*) (**Figure 3C**). Indeed, tamoxifen treatment reduced the tumor size in a durable manner (**Figure 3D**). Among ten tumors, 80% of the tumors completely regressed (regressed tumors), whereas 20% of the tumors did not respond (persistent tumors) (**Figure 3E**). Importantly, DE Tfh cells were absent in the regressed tumors, whereas persistent tumors still had abundant DE Tfh cells (**Figures 3F, G**).

We predicted that persisting tumors arose from DE Tfh cells that escaped Ezh2 gene deletion during the standard 5 daily tamoxifen gavage. To test this idea, we treated a group of tumor-bearing *Ezh2* deletable mice (*Roquin^san/+^;Cd4-cre ERT2 ^+/-^;Ezh2 ^f/f^*) with 3 daily tamoxifen gavage. Under this condition, we obtained only 50% tumor regression, leaving the other 50% of cases persisting (**Figure 3H**). After 4 weeks of tumor monitoring, regressed and persisting tumors were analyzed for EZH2 expression. As expected, persisting tumors had abundant DE Tfh cells (~ 10-20%) with intact EZH2 expression, whereas regressed tumors had greatly reduced levels of DE Tfh cells (~ 1-3%) with no EZH2 proteins (**Figures 3I, J**). This strongly suggests that persistent tumor growth is probably due to the residual DE Tfh cells that escaped the *Ezh2* gene deletion during the tamoxifen treatment.

### 3.3 *Ezh2* gene deletion decreases the proliferation of DE Tfh cells without inducing apoptosis

**Figure 4:**
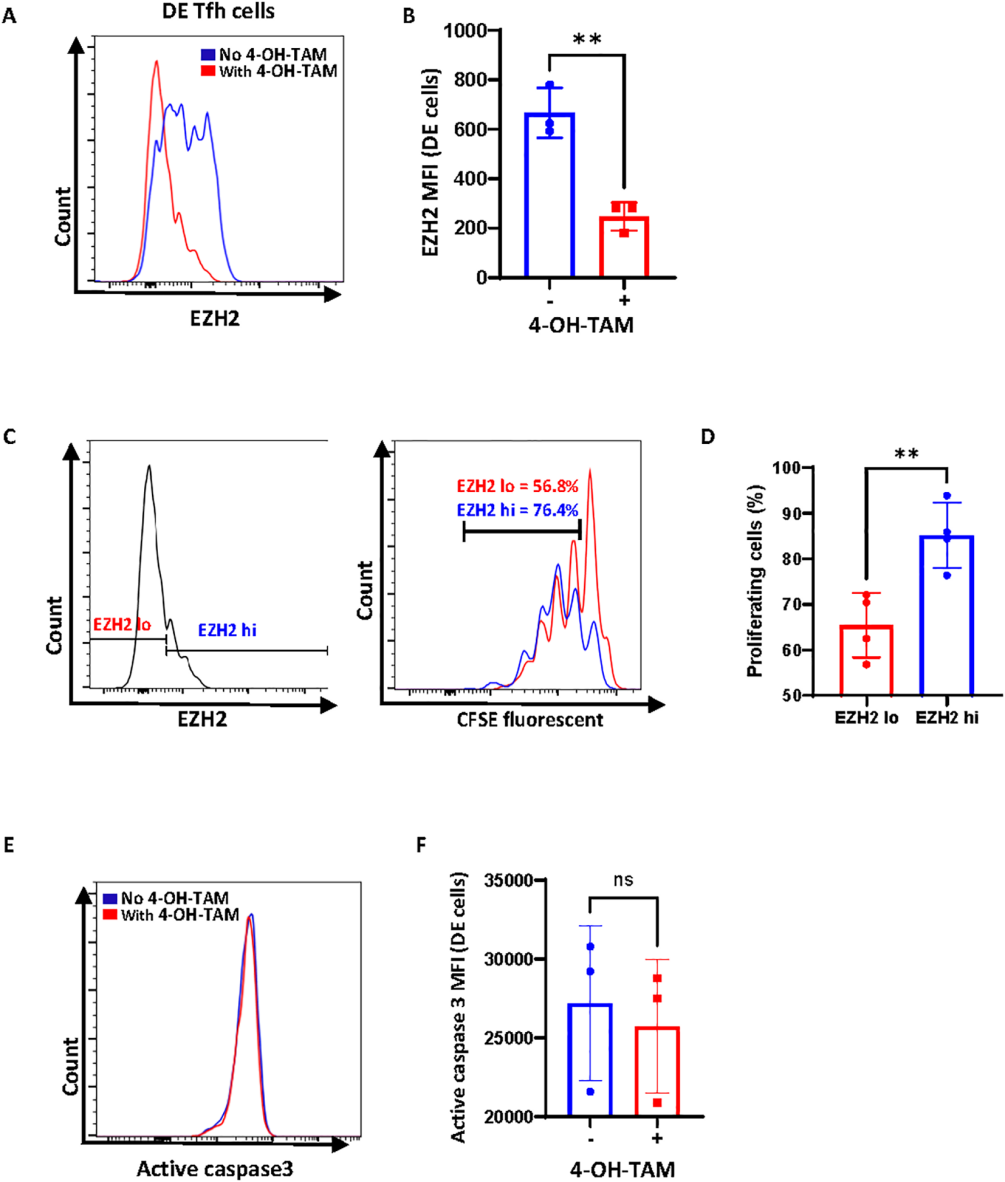
Acute Ezh2 gene deletion decreases the proliferation of DE Tfh cells without inducing apoptosis. **(A)** EZH2 depletion *in vitro*. Total CD4^+^ T cells were isolated from tumors grown in *Ezh2* conditional knockout mouse line (*Roquin^san/+^*; *Cd4-cre ERT2^+/-^*; *Ezh2^f/f^*) and cultured for 3 days *in vitro* without or with 4-OH-Tamoxifen as described in *Materials and methods*. A representative FACS plot of EZH2 levels in DE Tfh cells is shown. **(B)** Mean fluorescence intensity of EZH2 from three independent experiments. **(C)** CFSE dilution in EZH2^hi^ DE Tfh or EZH2^lo^ DE Tfh cells after treatment with 28nM 4-OH-Tamoxifen. **(D)** The percentage of proliferating cells in EZH2^hi^ DE Tfh vs EZH2^lo^ DE Tfh cells. **(E)** Representative histograms of active caspase 3 staining in DE Tfh cells. **(F)** Mean fluorescent intensity of active caspase 3 expression. Data are shown as the mean ± SD, **P < 0.01 (Student’s *t-*test).

Seeing that *Ezh2* gene deletion leads to tumor regression concomitant with the disappearance of DE Tfh cells *in vivo*, we predicted that DE Tfh cells may reduce proliferation or undergo apoptosis upon the loss of EZH2 expression. To test these possibilities, we set up an *in vitro* system in which tumor-derived CD4^+^ T cells from the *Ezh2* conditional knockout mouse line (*Roquin^san/+^;Cd4-cre ERT2^+/-^;Ezh2 ^f/f^*) grow in the absence or presence of 4-OH-tamoxifen. Under conditions in which ~90% of DE Tfh cells lose EZH2 protein (**Figures 4A, B**), we performed a CFSE dilution assay. As shown in **Figures 4C, D**, cells depleted of EZH2 proteins proliferated less compared to the cells still retaining EZH2 proteins. Under similar conditions, we could not detect any differences in the level of activated caspase 3 (**Figures 4E, F**). These data indicate that EZH2 is required for the sustained proliferation of DE Tfh cells but may not be directly linked to cell survival.

### 3.4 EZH2 inhibitor tazemetostat has anti-cancer efficacy

**Figure 5:**
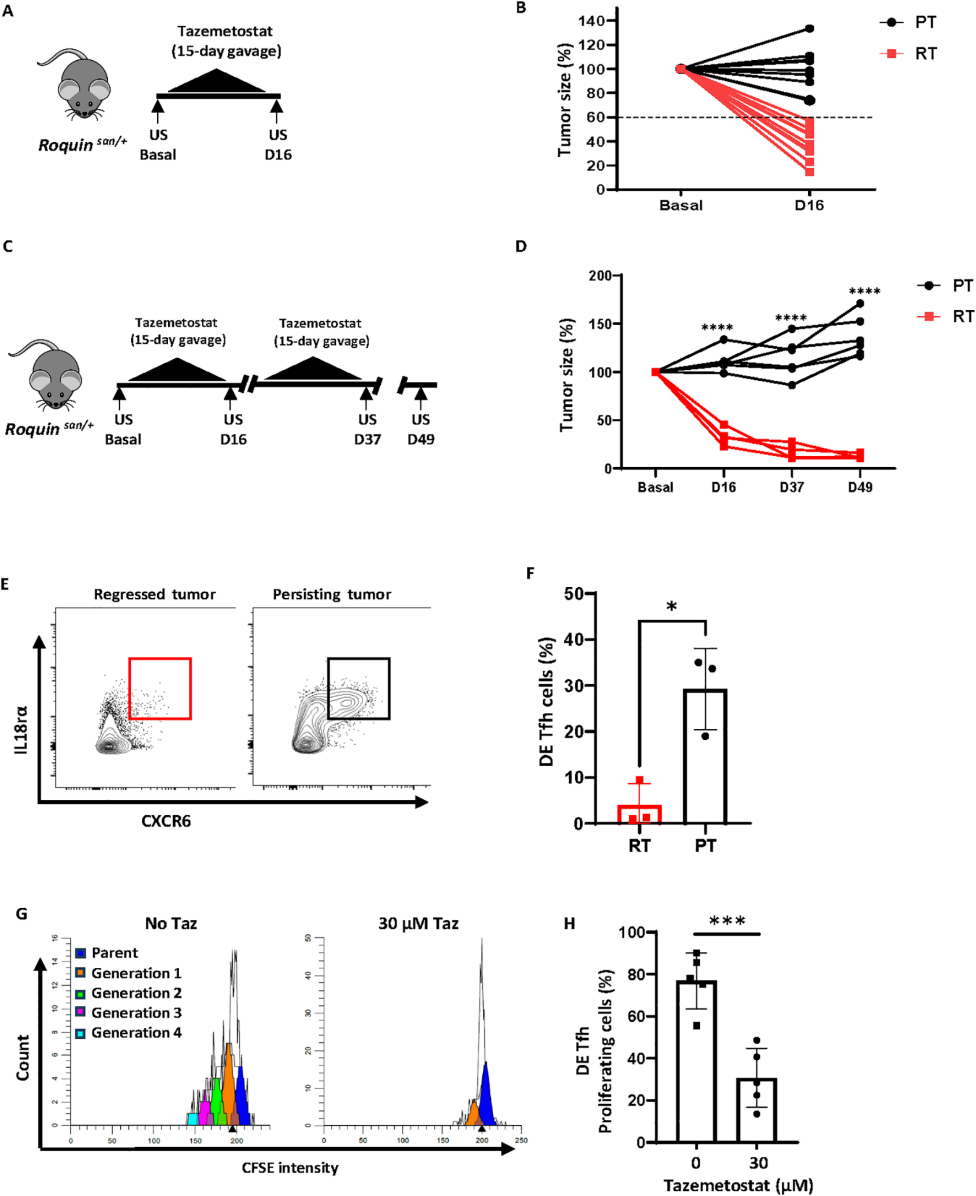
EZH2 inhibitor tazemetostat shows anti-cancer efficacy. **(A)** Experimental scheme (one round). Tumor-bearing *Roquin^san/+^* mice were treated with EZH2 inhibitor, tazemetostat, for 15 consecutive days. The tumor area was measured before and after treatment by ultrasound imaging. **(B)** Change of tumor area before (Basal; set as 100%) and after a 15-daily tazemetostat treatment (D16). Tumors with more than 40% reduction in area are considered as regressed tumors (RT, n=9) and those with less than 40% reduction as persisting tumors (PT, n=11). **(C)** Experimental scheme (two rounds). Tumor-bearing *Roquin^san/+^* mice were treated with EZH2 inhibitor, tazemetostat, for two 15-day cycles of tazemetostat with a 6-day rest in between. The tumor area was measured by ultrasound imaging at the indicated time points. **(D)** Change of tumor area before (Basal; set as 100%), after the first round (D16), and after the second round (D37 and D49). RT, n=4; PT, n=6. **(E, F)** DE Tfh cell profiling in regressed tumors (RT) versus persisting tumors (PT) by flow cytometry after two rounds of tazemetostat treatment (> D49). Representative FACS plots **(E)** and a summary of DE Tfh frequencies in RT and PT groups (n=3 per group) **(F)**. **(G, H)** CFSE dilution assay of FACS-sorted DE Tfh cells cultured for 3 days in the presence of IL-2 and IL-18 without or with tazemetostat. Representative FACS plots **(G)** and a summary of the frequencies of proliferating DE Tfh cells from five independent experiments **(H)**. Data are shown as the mean ± SD, *P < 0.05, ***P < 0.001 and ****P < 0.0001 [**(D)** Two-way ANOVA; **(F, H)** Student’s *t-*test].

Next, we tested whether tazemetostat (FDA-approved EZH2 inhibitor) has anti-cancer efficacy for AITL-like tumors. Among 20 tumors that were treated with tazemetostat (400 mg/kg; 15 daily gavage), nine tumors reduced their size by more than 40% (**Figures 5A, B**). Daily gavage for 15 consecutive days was harsh for mice that had tumors in the neck area, and we had to euthanize many of them after D16. However, ten mice remained healthy and were subjected to another round of 15-day tazemetostat treatment (**Figures 5C, D**). Four out of ten mice responded completely, whereas the other six tumors continued to grow even after the second round of tazemetostat treatment. Consistent with *Ezh2* gene deletion experiments, regressed tumors lacked DE Tfh cells, whereas persisting tumors contained abundant DE Tfh cells (**Figures 5E, F**). The partial response rate may be due to the limited delivery of bioactive tazemetostat *in vivo* since isolated DE Tfh cells were highly sensitive to tazemetostat *in vitro* (**Figures 5G, H**). Taken together, our data suggest that EZH2 inhibitors could be used to treat AITL.

### 3.5 Mouse DE Tfh gene signatures are enriched in a subset of AITL patient tumors

**Figure 6:**
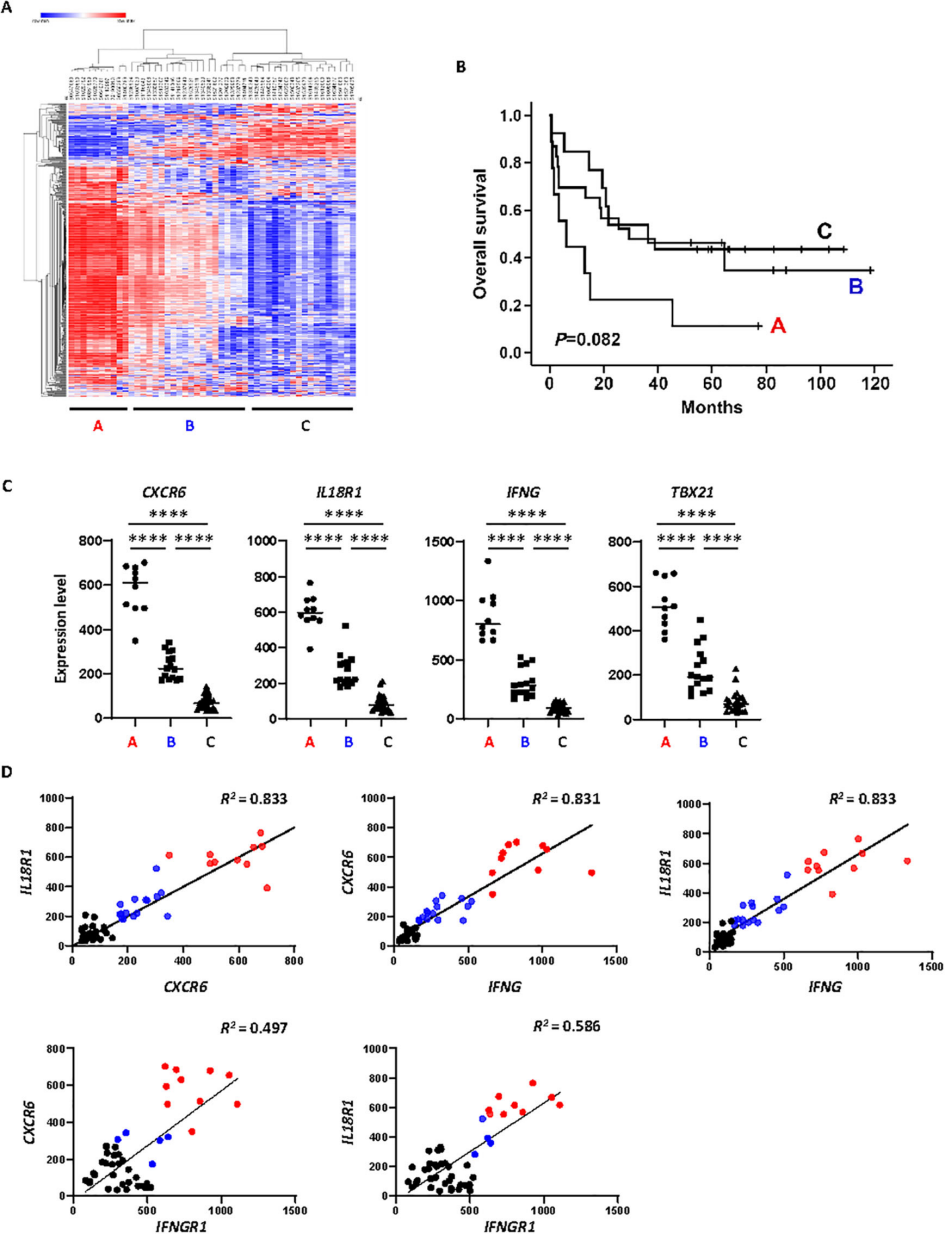
*Roquin^san/+^* DE Tfh gene signatures exist in a subset of AITL patient tumors. **(A)** Heatmap presentation of NanoString data obtained from 48 AITL biopsy samples. Each column represents patient ID (48); each row represents gene name (579). Samples were clustered into Subset A, B, and C according to gene expression profiles. Each gene was coded by the intensity and hue of the color from dark blue (lower) to dark red (higher). **(B)** Kaplan–Meier curves for overall survival stratified by Subet A, B, and **(C)** Overall survival did not differ significantly among the clusters (P = 0.082, log-rank test). **(C)** Differential expression patterns of *CXCR6*, *IL18R1*, *IFNG*, and *TBX21* in AITL subsets. ****P < 0.0001 (One-way ANOVA). **(D)** Simple linear regression test showed strong positive correlations between *CXCR6* and *IL18R1* expression levels (*R^2^* = 0.833), *CXCR6* and *IFNG* (*R^2^* = 0.831), as well as *IL18R1* and *IFNG* (*R^2^* = 0.833). In addition, expression levels between *CXCR6* and *IFNGR1* (*R^2^* = 0.497), and also *IL18R1* and *IFNGR1* (*R^2^* = 0.586) showed moderate correlations. Each dot represents individual patients belonging to Subset A (red), Subset B (blue), or Subset C (black).

Although Tfh cells expressing CXCR6 and IL-18R appear to be the major pathological cellular subset in our *Roquin^san/+^* mice, whether human AITL has a similar T cell subset is unknown. To address this question, we prepared RNA samples from excisional biopsy samples from 48 AITL patients and performed gene expression profiling using the NanoString nCounter Human Immunology V2 panel (579 immune signature genes). Heatmap analysis generated three subsets (**Figure 6A**). The overall survival curves of the three Subets did not differ significatly, although there was a trend toward reduced survaival in Subset A (**Figure 6B**). Intriguingly, genes involved in type I inflammation, *CXCR6*, *IL18R1, IFNG, and Tbx21* were highly expressed in Subset A (10/48), at intermediate levels in Subset B (15/48), and at lowest levels in Subset C (23/48) (**Figure 6C**). Congruent with the coexpression of *Cxcr6*, *Il18r1*, and *Ifng* genes in murine DE Tfh cells (**Figure 1C**, **Supplementary Figure 2**), we saw strong positive correlations between these three genes in human AITL samples (**Figure 6D**). Moreover, *CXCR6* and *IL18R1* expression levels showed moderate correlations with *IFNGR1* (**Figure 6D**). Based on these correlative data, we suggest that the *Roquin^san/+^* mouse model may represent Subset A of AITL patients. Subset A AITL tumors and *Roquin^san/+^* mouse tumors may have an IFN-γ-driven inflammatory microenvironment that polarizes Tfh cells towards Tfh1-like features. However, we could not detect any strong differences in disease prognosis or overall survival across the three AITL subtypes, suggesting that the presence of this type I inflammation may not be a strong prognostic indicator. Instead, the CXCR6 expression level and Tfh1-like features may be used to screen patients who may respond better to CXCR6 depletion or EZH2 inhibition.

Next, to support our NanoString data, we performed a meta-analysis of a publicly available dataset GSE51521 that used PTCL patient samples including 18 AITL cases. This study profiled gene expression patterns of PTCL samples using a human genome microarray chip (47, 000 transcripts) to discover molecular classifiers potentially blurring contrasts in low-abundance immune-related genes within the same subsets. Indeed, when we extracted AITL data and compared Z-scores, the differences were small (less than 4 standard deviations away from the mean of all the genes) for the type I inflammation genes *CXCR6*, *IL18R1*, *IFNG*, and *IFNGR1* (**Supplementary Figure 6A**). Despite these limitations, ~30% of AITL patients exhibited higher levels of *CXCR6*, *IL18R1*, *IFNG*, or *IFNGR1* than others. To further assess the relationships between these genes, we analyzed their expression correlations within the datasets. We observed moderate positive correlations between *CXCR6* and *IL18R1* (*R²* = 0.632) as well as *CXCR6* and *IFNG* (*R²* = 0.664) (**Supplementary Figure 6B**). Thus, ~30% of AITL samples showed elevated levels of CXCR6 and other Th1 markers in GSE51521, consistent with our NanoString data.

## 4 Discussion

Using single-cell transcriptome analysis and flow cytometry, we identified DE Tfh cells that express high levels of CXCR6 and IL-18 receptor in AITL-like tumors of *Roquin^san/+^* mice. DE Tfh cells are highly proliferative in an IL-18 and EZH2-dependent manner *in vitro* and have the capacity to expand B cells when adoptively transferred into lymphopenic recipients. Genetic or pharmacological inactivation of EZH2 activity reduced tumor burden concomitant with the disappearance of DE Tfh cells, indicating that DE Tfh cells are required for AITL tumor progression in mice. Our own gene expression profiling combined with meta-analysis of another AITL cohorts showed that ~20-30% of human AITL tumors have elevated levels of *CXCR6* and other type I inflammation signature suggesting the presence of DE Tfh counterparts in human AITL tumors.

An increased number of Tfh-like cells is a common feature of AITL in patients, as well as in multiple mouse models of AITL (1, 12, 13, 20, 21). However, it has been unclear whether there is a specialized Tfh-like subset that serves as the main driving force for the progression of AITL. In this study, we identified the CXCR6^+^IL-18R^+^EZH2^hi^ Tfh1-like subset (DE Tfh cells) as a potential neoplastic tumor-driving force. Two mouse models representing Tfh-derived peripheral T cell lymphomas have shown the presence of CD4^+^ T cell populations that share some of the DE Tfh characteristics. First, expression of an oncogenic Itk-Syk gene fusion construct (originally discovered in Tfh-derived follicular T cell lymphoma) in mice caused symptoms recapitulating human disease (37). Importantly, gene microarray experiments showed that CD4^+^ T cells in the Itk-Syk lymphoma upregulated genes encoding Ki-67, IFN-γ, CXCR6, and IL-18R1, hallmark features of DE Tfh cells (37). Second, overexpression of GAPDH in the T cell lineage has been shown to induce AITL-like disease in aged mice (38). RNA-seq data showed that GAPDH overexpression greatly elevated NF-kB signaling and accumulation of Tfh cells that express high levels of Ki-67 and EZH2 (38), features of DE Tfh cells and human peripheral T cell lymphoma (23, 24). However, these studies did not perform single-cell transcriptome analysis and, therefore, could not determine whether the changes in the gene expression levels reflect an outgrowth of a distinct tumor-driving Tfh subset. Nonetheless, these studies support our view that IFN-γ-driven inflammation and EZH2-dependent epigenetics promote the neoplastic transformation of Tfh cells and tumor growth in *Roquin^san/+^* mice.

DE Tfh cells express higher levels of EZH2, IL-18R, and CXCR6 than other Tfh cells. Each of these components may confer tumor-promoting capacities to DE Tfh cells. First, we showed that EZH2 is necessary to maintain the highly proliferative nature of DE Tfh cells. This was predictable considering that EZH2 promotes the differentiation and survival of Tfh cells, the cellular origin of AITL (25). Furthermore, among the CD4^+^ T cell subsets, IFN-γ producing Th1 cells were most reliant on EZH2 to sustain proliferation (39). Since DE Tfh cells have a hybrid feature of Tfh and Th1 cells, they could be particularly susceptible to EZH2 inhibition. We were unable to determine whether AITL patients with elevated EZH2 expression have worse disease progression or overall survival because EZH2 was not included in our NanoString panel. However, an independent study of 82 PTCL cases (including 10 AITL) reported that high EZH2 expression correlates with poor prognosis and reduced overall survival (24). Further studies are needed to validate EZH2 as a prognostic biomarker in AITL. Apart from PTCL, EZH2 is required for the sustained proliferation of B-cell lymphoma cells by downregulating cell cycle inhibitors (40). The FDA-approved EZH2 inhibitor tazemetostat is being used in the clinic to treat follicular lymphoma and other tumors (36, 41, 42), and new EZH2 inhibitors with improved pharmacological features are being developed (43). Second, IL-18 receptor signaling has been shown to induce IFN-γ production (44) and enhance T cell proliferation (33). This may allow DE Tfh cells to produce IFN-γ and maintain their highly proliferative status. However, our attempt to inhibit tumor growth by a systemic blockade of IL-18 has failed. We speculate that the current regimen (i.p. injection of mAb YIGIF74-1G7) is not sufficient to deplete IL-18 in the tumor microenvironment. Further work is required to test if targeting the IL-18 receptor or its downstream signaling via more effective reagents and/or delivery systems may reduce DE Tfh numbers and tumor growth. Lastly, CXCR6 and its ligand CXCL16 work together to promote cellular chemotaxis (when CXCL16 is secreted) or adhesion (when CXCL16 is membrane-bound) in many biological systems, including the immune system (45). For example, CXCR6-expressing T cells interact with CXCL16-expressing dendritic cells or epithelial cells for survival, expansion, and function (46, 47). Thus, CXCR6 may allow DE Tfh cells to migrate and survive in a niche where they drive B cell expansion. Further, we speculate that CXCR6 may promote the expression of IL-18 receptor such that DE Tfh cells persist. However, regardless of the role of CXCR6 in DE Tfh cell function, depletion of CXCR6-expressing cells using CXCR6 mAb may be a viable option for CXCR6-high AITL patients. Taken together, we predict that combinatorial targeting of EZH2 and CXCR6 may show synergistic therapeutic effects on AITL.

*Roquin^san/+^* mouse line was the first animal model of AITL (21, 48). Unlike later models, which reconstituted signature AITL mutations such as *TET2* and *RHOA*, the driving force of tumors arising in *Roquin^san/+^* mice remains less clear (12, 13, 20, 48). Despite this, *Roquin^san/+^* mice well recapitulate common features of Tfh-derived T cell lymphomas, such as hyperactive TCR signaling, increased Tfh specification, and definitive pathological manifestations (loss of T-B border and arborization of endothelial venules) in easily palpable tumors (1–2 superficial lymph nodes of 7–10 mm in diameter). These features allowed us to obtain enough material for single-cell analysis, which revealed the DE Tfh cluster. Using this *Roquin^san/+^* mouse model, we found evidence that ongoing T-B collaboration plays an important role in AITL tumor growth (14). This view was corroborated by findings that TET2-deficient B cells provide a niche for neoplastic Tfh-like cells in a *TET2-RHOA* mouse model (12). Our current study suggests that tumors grown in *Roquin^san/+^* mice may resemble ~20% of AITL cases in which IFN-γ-driven inflammation is prominent.

It is yet to be confirmed whether DE Tfh-like cells exist in human AITL. Analysis of our immune-focused NanoString data strongly indicates that CXCR6 and IL18R1 expression levels are highly correlated. Also, a microarray experiment indicated that ~30% of AITL patient samples express elevated levels of CXCR6. However, we had technical difficulties to identify CXCR6 and IL-18R1 proteins in our AITL FFPE samples since commercially available antibodies did not give sufficient signals. Further work is required to confirm CXCR6 and/or IL18R1 expression at the protein level in AITL patient samples. When immunohistochemistry of CXCR6/IL-18R1 becomes feasible, it would be important to compare AITL patient samples with non-malignant lymph nodes (such as tonsils) to determine if AITL cases belong to Subset C in our study express elevated levels of CXCR6 and/or IL18R1 compared with control Tfh cells. If it is the case, even Subset C AITL patients may benefit from DE Tfh depletion approches.

In sum, our work identified CXCR6^+^IL-18R^+^ DE Tfh cells as a uniquely expanded cellular subset with neoplastic nature in *Roquin^san/+^* mice. Depletion or persistence of DE Tfh cells highly correlated with regression or persistence of the tumors in *Roquin^san/+^* mice, suggesting that targeting DE Tfh cells in human AITL may be a promising therapy. We showed that some human AITL tumors are enriched with *CXCR6* and *IL-18R1*, suggesting that DE Tfh-like cells may exist in AITL and that targeting those cells may offer promising therapeutic options.

## 5 Conclusions

In the *Roquin^san/+^* mouse model of AITL, we identified a tumor-specific Tfh-like cellular subset, termed DE Tfh cells. DE Tfh cells express high levels of CXCR6, IL-18 receptor, and EZH2, and uniquely accumulate in tumors, but not in pre-tumor lymph nodes. These cells robustly proliferate in an EZH2 and IL-18-dependent manner *in vitro* and engrafted efficiently in lymphopenic mice. Thus, DE Tfh cells appear to be the neoplastic, tumor-driving component among the tumor-resident CD4^+^ T cell population. Importantly, the FDA-approved catalytic inhibitor of EZH2, tazemetostat, and anti-CXCR6 mAb showed promising anti-tumor efficacy against *Roquin^san/+^* AITL tumors with concomitant depletion of DE Tfh cells. Since ~20-30% of AITL patients show DE Tfh gene signatures, these patients may benefit from EZH2 inhibition or CXCR6 depletion treatments.

## Data Availability

The datasets presented in this article are available from the following repositories: *Roquin*^san/+^ mouse scRNA-Seq data from Gene Expression Omnibus (accession number GSE303738), https://www.ncbi.nlm.nih.gov/gds/?term=GSE303738; AITL patients’ NanoString nCounter data from zenodo (accession number 18308545), https://zenodo.org/records/18308545.

## References

[1] Lage L Culler HF Reichert CO da Siqueira SAC Pereira J . Angioimmunoblastic T-cell lymphoma and correlated neoplasms with T-cell follicular helper phenotype: from molecular mechanisms to therapeutic advances. Front Oncol. (2023) 13:1177590. doi: 10.3389/fonc.2023.1177590, PMID: 37182145 PMC10169672

[2] Attygalle AD Kyriakou C Dupuis J Grogg KL Diss TC Wotherspoon AC . Histologic evolution of angioimmunoblastic T-cell lymphoma in consecutive biopsies: clinical correlation and insights into natural history and disease progression. Am J Surg Pathol. (2007) 31:1077–88. doi: 10.1097/PAS.0b013e31802d68e9, PMID: 17592275

[3] de Leval L Rickman DS Thielen C Reynies A Huang YL Delsol G . The gene expression profile of nodal peripheral T-cell lymphoma demonstrates a molecular link between angioimmunoblastic T-cell lymphoma (AITL) and follicular helper T (TFH) cells. Blood. (2007) 109:4952–63. doi: 10.1182/blood-2006-10-055145, PMID: 17284527

[4] Yu H Shahsafaei A Dorfman DM . Germinal-center T-helper-cell markers PD-1 and CXCL13 are both expressed by neoplastic cells in angioimmunoblastic T-cell lymphoma. Am J Clin Pathol. (2009) 131:33–41. doi: 10.1309/AJCP62WRKERPXDRT, PMID: 19095563

[5] Zhan HQ Li XQ Zhu XZ Lu HF Zhou XY Chen Y . Expression of follicular helper T cell markers in nodal peripheral T cell lymphomas: a tissue microarray analysis of 162 cases. J Clin Pathol. (2011) 64:319–24. doi: 10.1136/jcp.2010.084459, PMID: 21330314

[6] Alaggio R Amador C Anagnostopoulos I Attygalle AD Araujo IBO Berti E . The 5th edition of the world health organization classification of haematolymphoid tumours: lymphoid neoplasms. Leukemia. (2022) 36:1720–48. doi: 10.1038/s41375-022-01620-2, PMID: 35732829 PMC9214472

[7] Dobay MP Lemonnier F Missiaglia E Bastard C Vallois D Jais JP . Integrative clinicopathological and molecular analyses of angioimmunoblastic T-cell lymphoma and other nodal lymphomas of follicular helper T-cell origin. Haematologica. (2017) 102:e148–51. doi: 10.3324/haematol.2016.158428, PMID: 28082343 PMC5395128

[8] Zhang Q Yin L Lai Q Zhao Y Peng H . Advances in the pathogenesis and therapeutic strategies of angioimmunoblastic T-cell lymphoma. Clin Exp Med. (2023) 23:4219–35. doi: 10.1007/s10238-023-01197-9, PMID: 37759042

[9] Crotty S . T follicular helper cell biology: A decade of discovery and diseases. Immunity. (2019) 50:1132–48. doi: 10.1016/j.immuni.2019.04.011, PMID: 31117010 PMC6532429

[10] Kumar S Basto AP Ribeiro F Almeida SCP Campos P Peres C . Specialized Tfh cell subsets driving type-1 and type-2 humoral responses in lymphoid tissue. Cell Discov. (2024) 10:64. doi: 10.1038/s41421-024-00681-0, PMID: 38834551 PMC11150427

[11] Fazilleau N Mark L McHeyzer-Williams LJ McHeyzer-Williams MG . Follicular helper T cells: lineage and location. Immunity. (2009) 30:324–35. doi: 10.1016/j.immuni.2009.03.003, PMID: 19303387 PMC2731675

[12] Fujisawa M Nguyen TB Abe Y Suehara Y Fukumoto K Suma S . Clonal germinal center B cells function as a niche for T-cell lymphoma. Blood. (2022) 140:1937–50. doi: 10.1182/blood.2022015451, PMID: 35921527 PMC10653021

[13] Leca J Lemonnier F Meydan C Foox J El Ghamrasni S Mboumba DL . IDH2 and TET2 mutations synergize to modulate T Follicular Helper cell functional interaction with the AITL microenvironment. Cancer Cell. (2023) 41:323–339.e310. doi: 10.1016/j.ccell.2023.01.003, PMID: 36736318

[14] Witalis M Chang J Zhong MC Bouklouch Y Panneton V Li J . Progression of AITL-like tumors in mice is driven by Tfh signature proteins and T-B cross talk. Blood Adv. (2020) 4:868–79. doi: 10.1182/bloodadvances.2019001114, PMID: 32130407 PMC7065475

[15] Yoo HY Sung MK Lee SH Kim S Lee H Park S . A recurrent inactivating mutation in RHOA GTPase in angioimmunoblastic T cell lymphoma. Nat Genet. (2014) 46:371–5. doi: 10.1038/ng.2916, PMID: 24584070

[16] Sakata-Yanagimoto M Enami T Yoshida K Shiraishi Y Ishii R Miyake Y . Somatic RHOA mutation in angioimmunoblastic T cell lymphoma. Nat Genet. (2014) 46:171–5. doi: 10.1038/ng.2872, PMID: 24413737

[17] Palomero T Couronne L Khiabanian H Kim MY Ambesi-Impiombato A Perez-Garcia A . Recurrent mutations in epigenetic regulators, RHOA and FYN kinase in peripheral T cell lymphomas. Nat Genet. (2014) 46:166–70. doi: 10.1038/ng.2873, PMID: 24413734 PMC3963408

[18] Yao WQ Wu F Zhang W Chuang SS Thompson JS Chen Z . Angioimmunoblastic T-cell lymphoma contains multiple clonal T-cell populations derived from a common TET2 mutant progenitor cell. J Pathol. (2020) 250:346–57. doi: 10.1002/path.5376, PMID: 31859368 PMC7064999

[19] Vallois D Dobay MP Morin RD Lemonnier F Missiaglia E Juilland M . Activating mutations in genes related to TCR signaling in angioimmunoblastic and other follicular helper T-cell-derived lymphomas. Blood. (2016) 128:1490–502. doi: 10.1182/blood-2016-02-698977, PMID: 27369867

[20] Cortes JR Ambesi-Impiombato A Couronne L Quinn SA Kim CS da Silva Almeida AC . RHOA G17V induces T follicular helper cell specification and promotes lymphomagenesis. Cancer Cell. (2018) 33:259–273.e257. doi: 10.1016/j.ccell.2018.01.001, PMID: 29398449 PMC5811310

[21] Ellyard JI Chia T Rodriguez-Pinilla SM Martin JL Hu X Navarro-Gonzalez M . Heterozygosity for Roquinsan leads to angioimmunoblastic T-cell lymphoma-like tumors in mice. Blood. (2012) 120:812–21. doi: 10.1182/blood-2011-07-365130, PMID: 22700722

[22] Lee SK Silva DG Martin JL Pratama A Hu X Chang PP . Interferon-gamma excess leads to pathogenic accumulation of follicular helper T cells and germinal centers. Immunity. (2012) 37:880–92. doi: 10.1016/j.immuni.2012.10.010, PMID: 23159227

[23] Chai J Choudhuri J Gong JZ Wang Y Tian X . Upregulation of enhancer of zeste homolog 2 (EZH2) with associated pERK co-expression and PRC2 complex protein SUZ12 correlation in adult T-cell leukemia/lymphoma. Cancers (Basel). (2024) 16. doi: 10.3390/cancers16030646, PMID: 38339397 PMC10854612

[24] Zhang H Lv H Jia X Hu G Kong L Zhang T . Clinical significance of enhancer of zeste homolog 2 and histone deacetylases 1 and 2 expression in peripheral T-cell lymphoma. Oncol Lett. (2019) 18:1415–23. doi: 10.3892/ol.2019.10410, PMID: 31423206 PMC6607380

[25] Li F Zeng Z Xing S Gullicksrud JA Shan Q Choi J . Ezh2 programs TFH differentiation by integrating phosphorylation-dependent activation of Bcl6 and polycomb-dependent repression of p19Arf. Nat Commun. (2018) 9:5452. doi: 10.1038/s41467-018-07853-z, PMID: 30575739 PMC6303346

[26] Sledzinska A Hemmers S Mair F Gorka O Ruland J Fairbairn L . TGF-beta signalling is required for CD4(+) T cell homeostasis but dispensable for regulatory T cell function. PLoS Biol. (2013) 11:e1001674. doi: 10.1371/journal.pbio.1001674, PMID: 24115907 PMC3792861

[27] Stuart T Butler A Hoffman P Hafemeister C Papalexi E Mauck WM . Comprehensive integration of single-cell data. Cell. (2019) 177:1888–1902.e1821. doi: 10.1016/j.cell.2019.05.031, PMID: 31178118 PMC6687398

[28] Waltman L van Eck NJ . A smart local moving algorithm for large-scale modularity-based community detection. Eur Phys J B. (2013) 86. doi: 10.1140/epjb/e2013-40829-0

[29] McInnes L Healy J Melville J . UMAP: Uniform manifold approximation and projection for dimension reduction. arXiv. (2020). doi: 10.48550/arXiv.1802.03426

[30] Waggott D Chu K Yin S Wouters BG Liu FF Boutros PC . NanoStringNorm: an extensible R package for the pre-processing of NanoString mRNA and miRNA data. Bioinformatics. (2012) 28:1546–8. doi: 10.1093/bioinformatics/bts188, PMID: 22513995 PMC3356845

[31] Robinson MD McCarthy DJ Smyth GK . edgeR: a Bioconductor package for differential expression analysis of digital gene expression data. Bioinformatics. (2010) 26:139–40. doi: 10.1093/bioinformatics/btp616, PMID: 19910308 PMC2796818

[32] Zhu M Li N Fan L Wu R Cao L Ren Y . Single-cell transcriptomic and spatial analysis reveal the immunosuppressive microenvironment in relapsed/refractory angioimmunoblastic T-cell lymphoma. Blood Cancer J. (2024) 14:218. doi: 10.1038/s41408-024-01199-0, PMID: 39695118 PMC11655871

[33] Almutairi SM Ali AK He W Yang DS Ghorbani P Wang L . Interleukin-18 up-regulates amino acid transporters and facilitates amino acid-induced mTORC1 activation in natural killer cells. J Biol Chem. (2019) 294:4644–55. doi: 10.1074/jbc.RA118.005892, PMID: 30696773 PMC6433059

[34] Liu Q Lee JH Kang HM Kim CH . Identification of the niche and mobilization mechanism for tissue-protective multipotential bone marrow ILC progenitors. Sci Adv. (2022) 8:eabq1551. doi: 10.1126/sciadv.abq1551, PMID: 36417511 PMC9683709

[35] Wang B Wang Y Sun X Deng G Huang W Wu X . CXCR6 is required for antitumor efficacy of intratumoral CD8(+) T cell. J Immunother Cancer. (2021) 9. doi: 10.1136/jitc-2021-003100, PMID: 34462326 PMC8407215

[36] Morin RD Arthur SE Assouline S . Treating lymphoma is now a bit EZ-er. Blood Adv. (2021) 5:2256–63. doi: 10.1182/bloodadvances.2020002773, PMID: 33904892 PMC8095133

[37] Jaeger A Gambheer SMM Sun X Chernyakov D Skorobohatko O Mack T . Activated granulocytes and inflammatory cytokine signaling drive T-cell lymphoma progression and disease symptoms. Blood. (2023) 141:2824–40. doi: 10.1182/blood.2022015653, PMID: 36696631

[38] Mondragon L Mhaidly R De Donatis GM Tosolini M Dao P Martin AR . GAPDH overexpression in the T cell lineage promotes angioimmunoblastic T cell lymphoma through an NF-kappaB-dependent mechanism. Cancer Cell. (2019) 36:268–287.e210. doi: 10.1016/j.ccell.2019.07.008, PMID: 31447347

[39] Yang XP Jiang K Hirahara K Vahedi G Afzali B Sciume G . EZH2 is crucial for both differentiation of regulatory T cells and T effector cell expansion. Sci Rep. (2015) 5:10643. doi: 10.1038/srep10643, PMID: 26090605 PMC4473539

[40] Beguelin W Teater M Gearhart MD Calvo Fernandez MT Goldstein RL Cardenas MG . EZH2 and BCL6 cooperate to assemble CBX8-BCOR complex to repress bivalent promoters, mediate germinal center formation and lymphomagenesis. Cancer Cell. (2016) 30:197–213. doi: 10.1016/j.ccell.2016.07.006, PMID: 27505670 PMC5000552

[41] Tong KI Yoon S Isaev K Bakhtiari M Lackraj T He MY . Combined EZH2 inhibition and IKAROS degradation leads to enhanced antitumor activity in diffuse large B-cell lymphoma. Clin Cancer Res. (2021) 27:5401–14. doi: 10.1158/1078-0432.CCR-20-4027, PMID: 34168051

[42] Chen F Liu J Song X DuCote TJ Byrd AL Wang C . EZH2 inhibition confers PIK3CA-driven lung tumors enhanced sensitivity to PI3K inhibition. Cancer Lett. (2022) 524:151–60. doi: 10.1016/j.canlet.2021.10.010, PMID: 34655667 PMC8743034

[43] Izutsu K Makita S Nosaka K Yoshimitsu M Utsunomiya A Kusumoto S . An open-label, single-arm phase 2 trial of valemetostat for relapsed or refractory adult T-cell leukemia/lymphoma. Blood. (2023) 141:1159–68. doi: 10.1182/blood.2022016862, PMID: 36150143 PMC10651775

[44] Okamura H Tsutsi H Komatsu T Yutsudo M Hakura A Tanimoto T . Cloning of a new cytokine that induces IFN-gamma production by T cells. Nature. (1995) 378:88–91. doi: 10.1038/378088a0, PMID: 7477296

[45] Korbecki J Bajdak-Rusinek K Kupnicka P Kapczuk P Siminska D Chlubek D . The role of CXCL16 in the pathogenesis of cancer and other diseases. Int J Mol Sci. (2021) 22. doi: 10.3390/ijms22073490, PMID: 33800554 PMC8036711

[46] Di Pilato M Kfuri-Rubens R Pruessmann JN Ozga AJ Messemaker M Cadilha BL . CXCR6 positions cytotoxic T cells to receive critical survival signals in the tumor microenvironment. Cell. (2021) 184:4512–4530.e4522. doi: 10.1016/j.cell.2021.07.015, PMID: 34343496 PMC8719451

[47] Hase K Murakami T Takatsu H Shimaoka T Iimura M Hamura K . The membrane-bound chemokine CXCL16 expressed on follicle-associated epithelium and M cells mediates lympho-epithelial interaction in GALT. J Immunol. (2006) 176:43–51. doi: 10.4049/jimmunol.176.1.43, PMID: 16365394

[48] Mhaidly R Krug A Gaulard P Lemonnier F Ricci JE Verhoeyen E . New preclinical models for angioimmunoblastic T-cell lymphoma: filling the GAP. Oncogenesis. (2020) 9:73. doi: 10.1038/s41389-020-00259-x, PMID: 32796826 PMC7427806

