# Supplementary material for: Identification of a neoplastic Tfh-like cellular subset in a mouse model of angioimmunoblastic T cell lymphoma
Source: Front Oncol. 2026 Jan 30;16:1715613. doi: 10.3389/fonc.2026.1715613 (PMC12900724; doi:10.3389/fonc.2026.1715613)
Supplement: Supplementary file 1 [file Presentation1.pptx]

## Slide 1
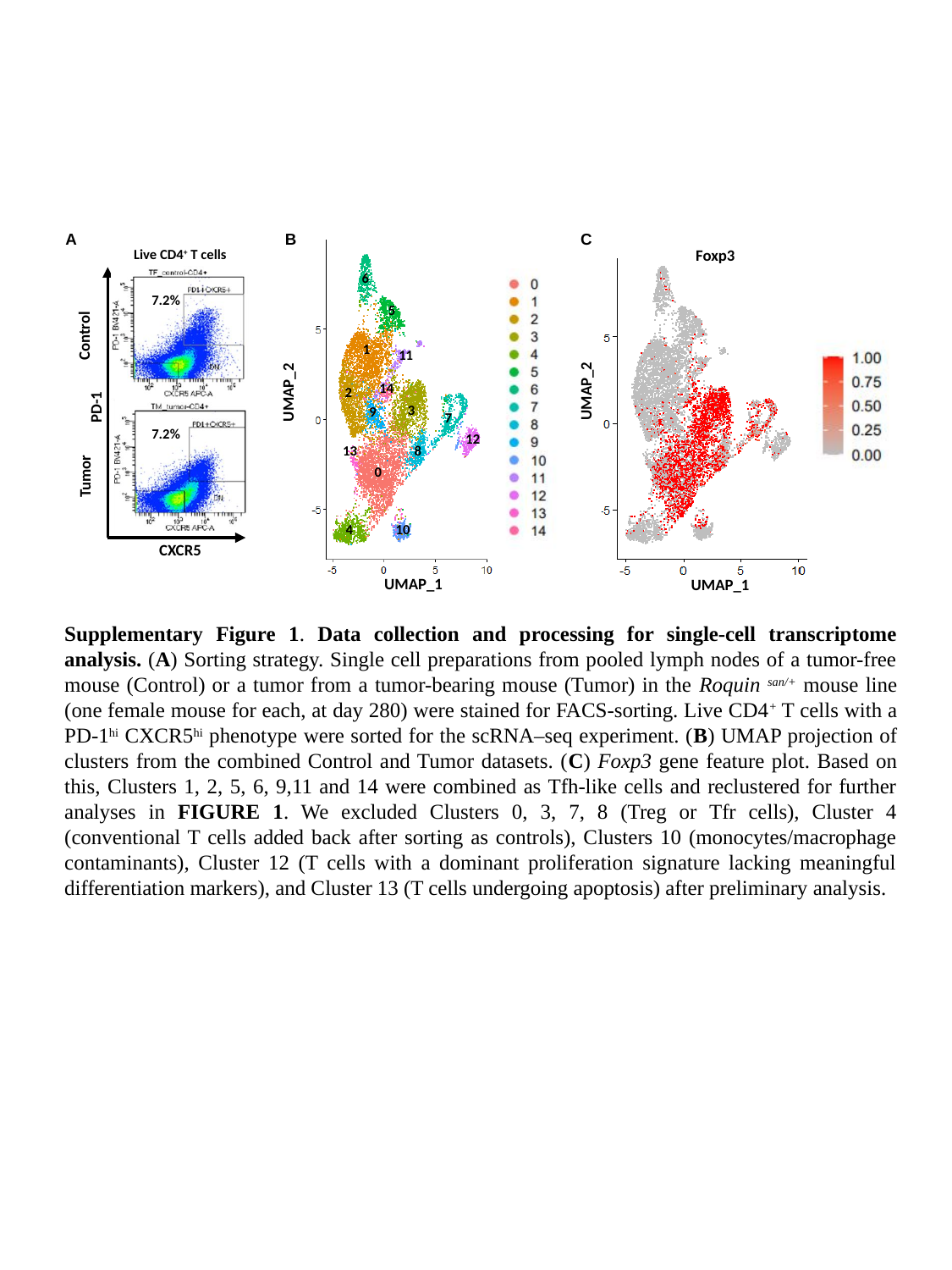

A
B
C
Foxp3
Live CD4+ T cells
6
7.2%
5
Control
1
11
14
UMAP_2
UMAP_2
2
PD-1
3
9
7
7.2%
12
13
8
0
Tumor
10
4
CXCR5
UMAP_1
UMAP_1
Supplementary Figure 1. Data collection and processing for single-cell transcriptome analysis. (A) Sorting strategy. Single cell preparations from pooled lymph nodes of a tumor-free mouse (Control) or a tumor from a tumor-bearing mouse (Tumor) in the Roquin san/+ mouse line (one female mouse for each, at day 280) were stained for FACS-sorting. Live CD4+ T cells with a PD-1hi CXCR5hi phenotype were sorted for the scRNA–seq experiment. (B) UMAP projection of clusters from the combined Control and Tumor datasets. (C) Foxp3 gene feature plot. Based on this, Clusters 1, 2, 5, 6, 9,11 and 14 were combined as Tfh-like cells and reclustered for further analyses in FIGURE 1. We excluded Clusters 0, 3, 7, 8 (Treg or Tfr cells), Cluster 4 (conventional T cells added back after sorting as controls), Clusters 10 (monocytes/macrophage contaminants), Cluster 12 (T cells with a dominant proliferation signature lacking meaningful differentiation markers), and Cluster 13 (T cells undergoing apoptosis) after preliminary analysis.

## Slide 2
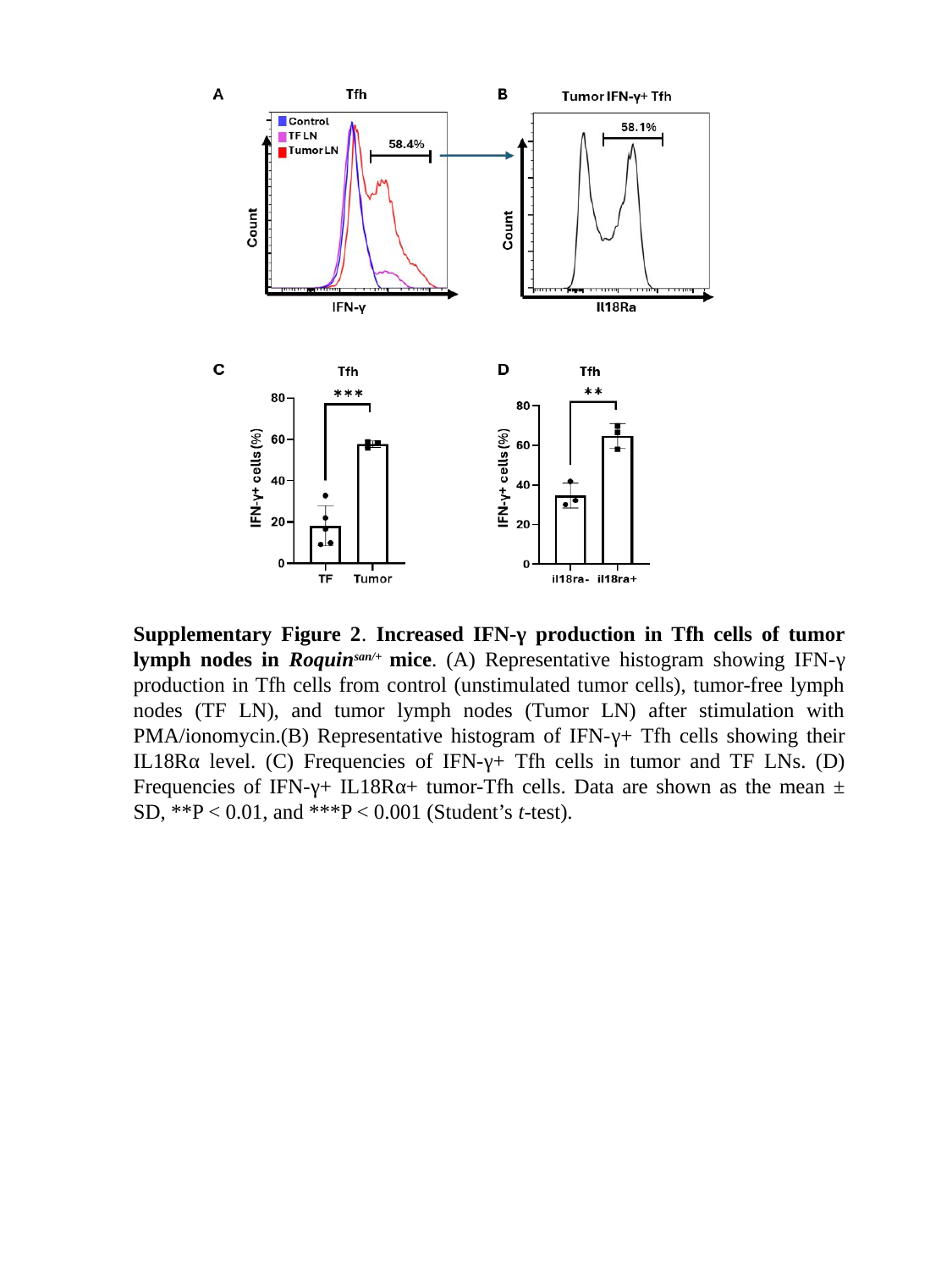

Supplementary Figure 2. Increased IFN-γ production in Tfh cells of tumor lymph nodes in Roquinsan/+ mice. (A) Representative histogram showing IFN-γ production in Tfh cells from control (unstimulated tumor cells), tumor-free lymph nodes (TF LN), and tumor lymph nodes (Tumor LN) after stimulation with PMA/ionomycin.(B) Representative histogram of IFN-γ+ Tfh cells showing their IL18Rα level. (C) Frequencies of IFN-γ+ Tfh cells in tumor and TF LNs. (D) Frequencies of IFN-γ+ IL18Rα+ tumor-Tfh cells. Data are shown as the mean ± SD, **P < 0.01, and ***P < 0.001 (Student’s t-test).

## Slide 3
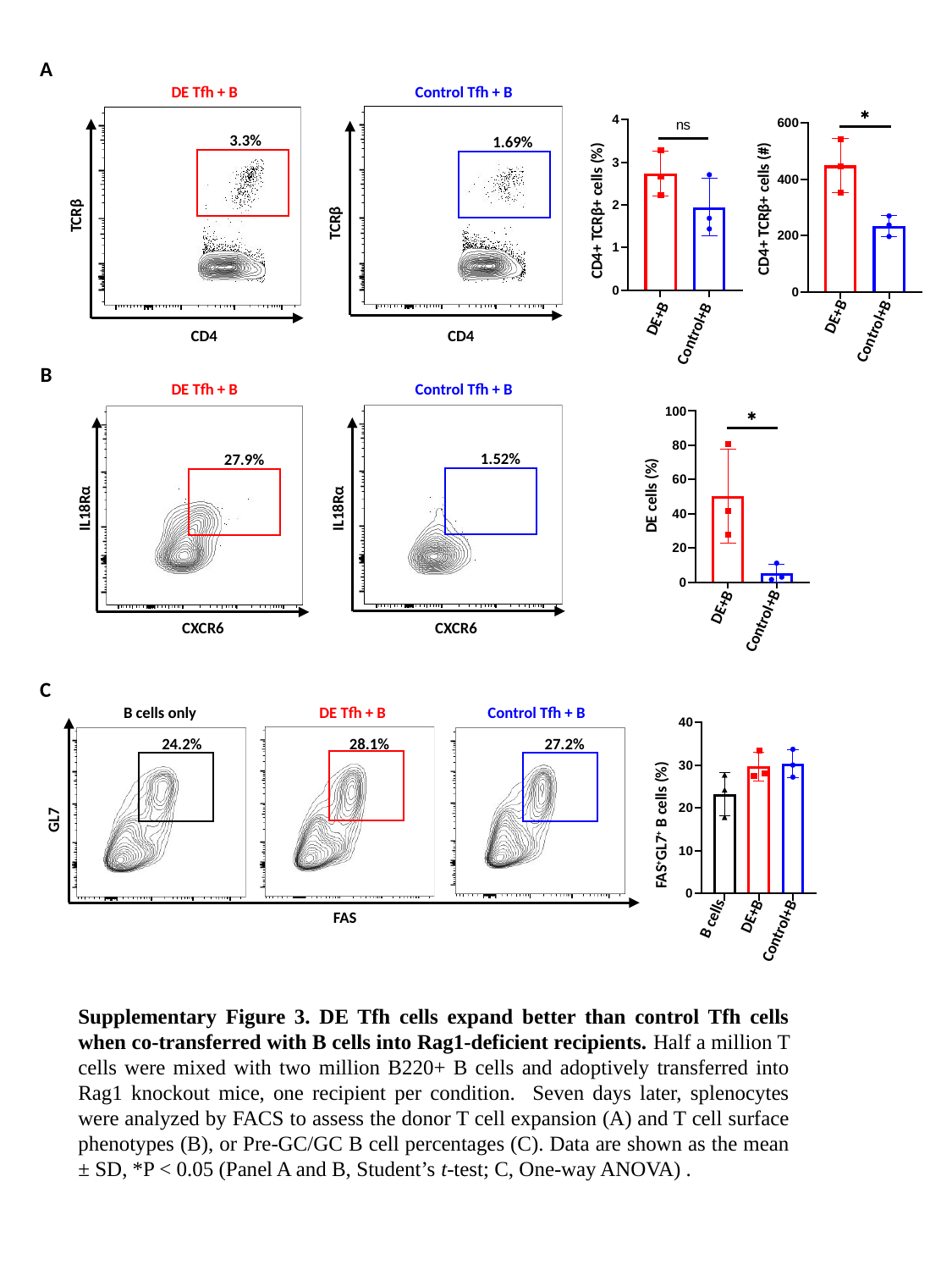

A
DE Tfh + B
Control Tfh + B
3.3%
1.69%
CD4+ TCRβ+ cells (#)
CD4+ TCRβ+ cells (%)
TCRβ
TCRβ
DE+B
DE+B
Control+B
Control+B
CD4
CD4
B
DE Tfh + B
Control Tfh + B
DE cells (%)
DE+B
Control+B
1.52%
27.9%
IL18Rα
IL18Rα
CXCR6
CXCR6
C
DE+B
B cells
Control+B
B cells only
DE Tfh + B
Control Tfh + B
27.2%
24.2%
28.1%
GL7
FAS+GL7+ B cells (%)
FAS
Supplementary Figure 3. DE Tfh cells expand better than control Tfh cells when co-transferred with B cells into Rag1-deficient recipients. Half a million T cells were mixed with two million B220+ B cells and adoptively transferred into Rag1 knockout mice, one recipient per condition. Seven days later, splenocytes were analyzed by FACS to assess the donor T cell expansion (A) and T cell surface phenotypes (B), or Pre-GC/GC B cell percentages (C). Data are shown as the mean ± SD, *P < 0.05 (Panel A and B, Student’s t-test; C, One-way ANOVA) .

## Slide 4
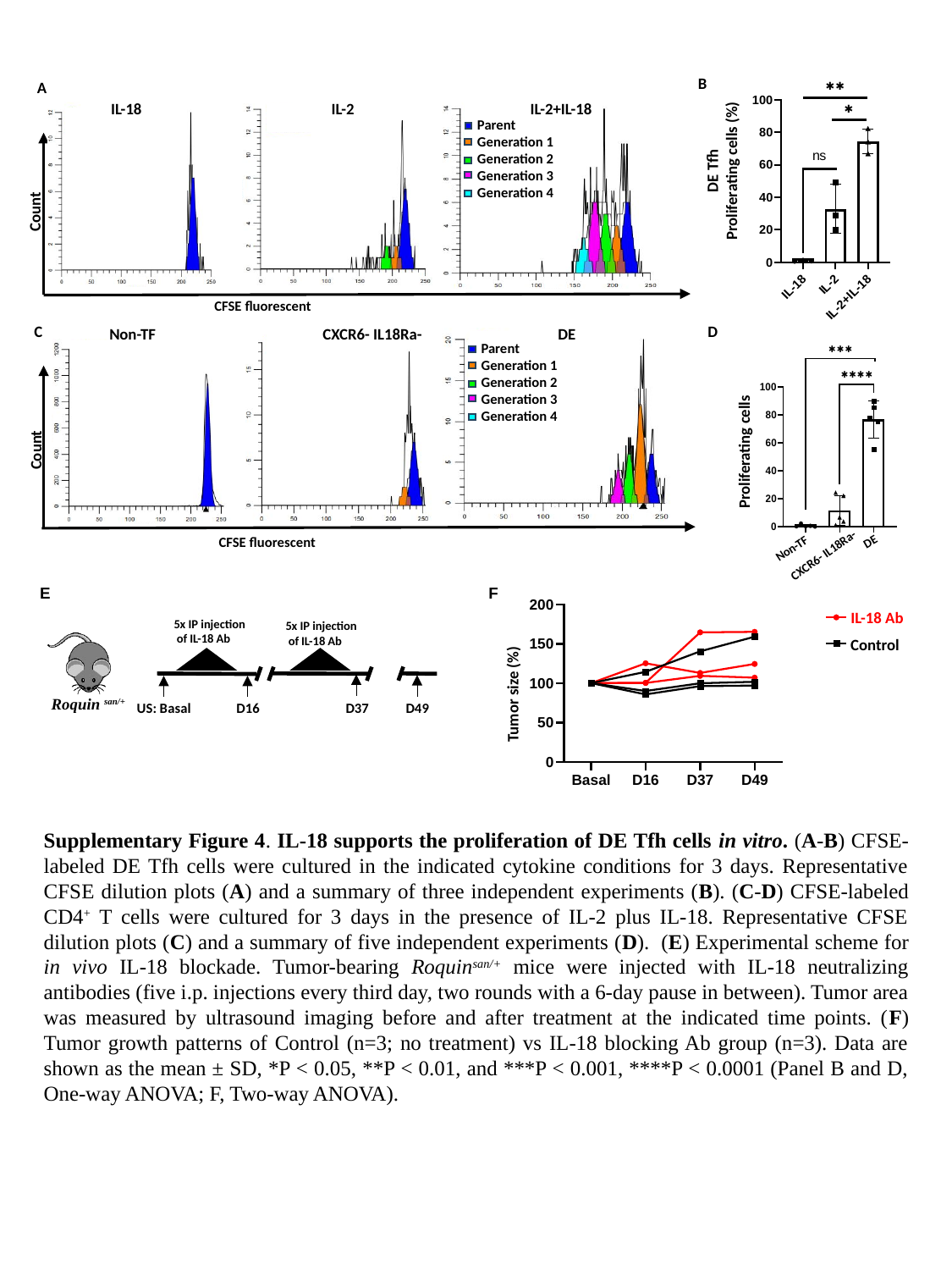

B
A
IL-18
IL-2
IL-2+IL-18
Parent
Generation 1
Generation 2
Generation 3
Generation 4
DE Tfh
Proliferating cells (%)
Count
CFSE fluorescent
D
C
Non-TF
CXCR6- IL18Ra-
DE
Parent
Generation 1
Generation 2
Generation 3
Generation 4
Proliferating cells
Count
CFSE fluorescent
DE
Non-TF
CXCR6- IL18Ra-
E
F
IL-18 Ab
5x IP injection
 of IL-18 Ab
5x IP injection
 of IL-18 Ab
Control
Tumor size (%)
Roquin san/+
US: Basal
D16
D37
D49
Supplementary Figure 4. IL-18 supports the proliferation of DE Tfh cells in vitro. (A-B) CFSE-labeled DE Tfh cells were cultured in the indicated cytokine conditions for 3 days. Representative CFSE dilution plots (A) and a summary of three independent experiments (B). (C-D) CFSE-labeled CD4+ T cells were cultured for 3 days in the presence of IL-2 plus IL-18. Representative CFSE dilution plots (C) and a summary of five independent experiments (D). (E) Experimental scheme for in vivo IL-18 blockade. Tumor-bearing Roquinsan/+ mice were injected with IL-18 neutralizing antibodies (five i.p. injections every third day, two rounds with a 6-day pause in between). Tumor area was measured by ultrasound imaging before and after treatment at the indicated time points. (F) Tumor growth patterns of Control (n=3; no treatment) vs IL-18 blocking Ab group (n=3). Data are shown as the mean ± SD, *P < 0.05, **P < 0.01, and ***P < 0.001, ****P < 0.0001 (Panel B and D, One-way ANOVA; F, Two-way ANOVA).

## Slide 5
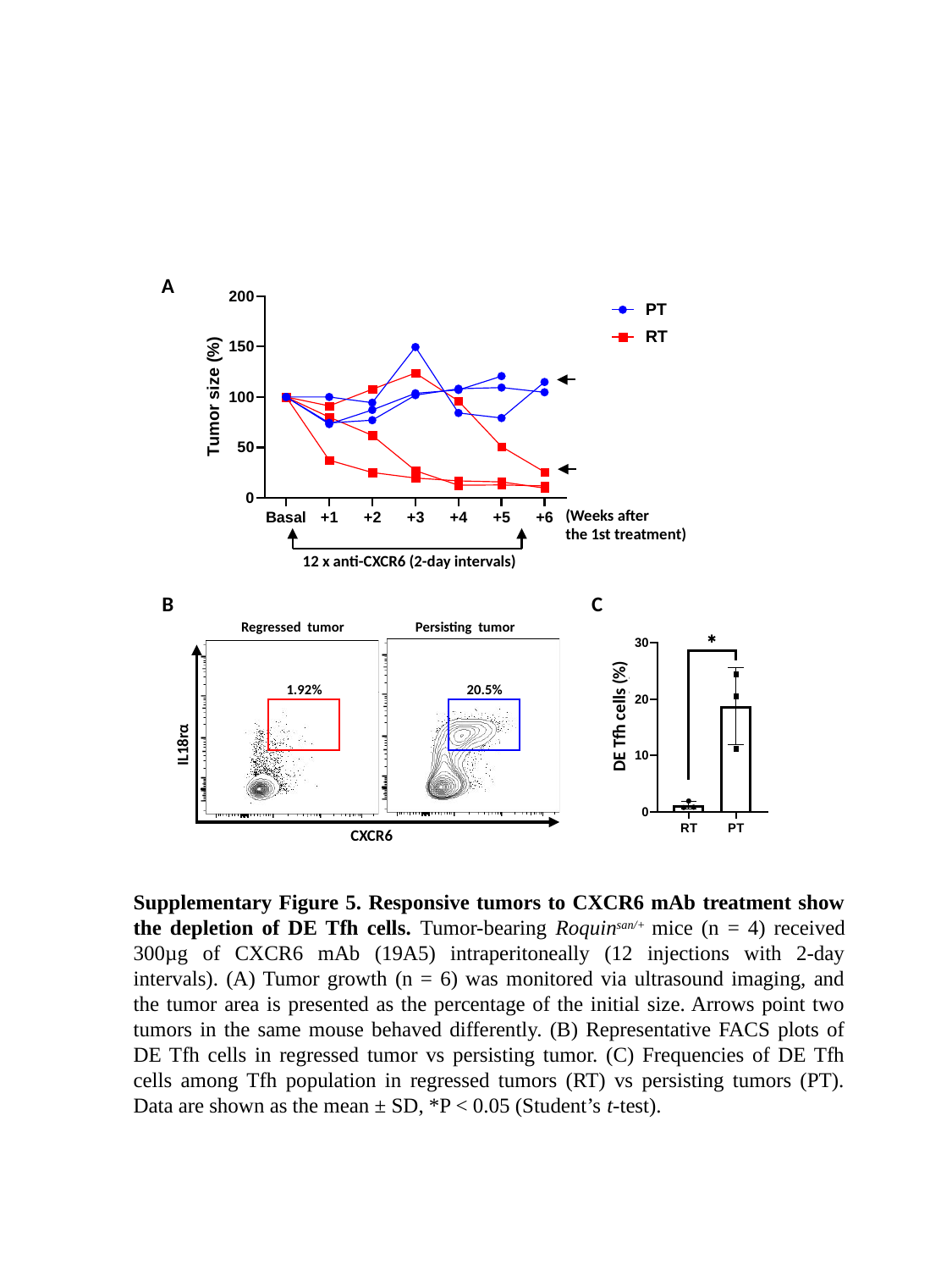

A
(Weeks after
the 1st treatment)
12 x anti-CXCR6 (2-day intervals)
B
Regressed tumor
Persisting tumor
1.92%
20.5%
IL18rα
CXCR6
C
DE Tfh cells (%)
Supplementary Figure 5. Responsive tumors to CXCR6 mAb treatment show the depletion of DE Tfh cells. Tumor-bearing Roquinsan/+ mice (n = 4) received 300µg of CXCR6 mAb (19A5) intraperitoneally (12 injections with 2-day intervals). (A) Tumor growth (n = 6) was monitored via ultrasound imaging, and the tumor area is presented as the percentage of the initial size. Arrows point two tumors in the same mouse behaved differently. (B) Representative FACS plots of DE Tfh cells in regressed tumor vs persisting tumor. (C) Frequencies of DE Tfh cells among Tfh population in regressed tumors (RT) vs persisting tumors (PT). Data are shown as the mean ± SD, *P < 0.05 (Student’s t-test).

## Slide 6
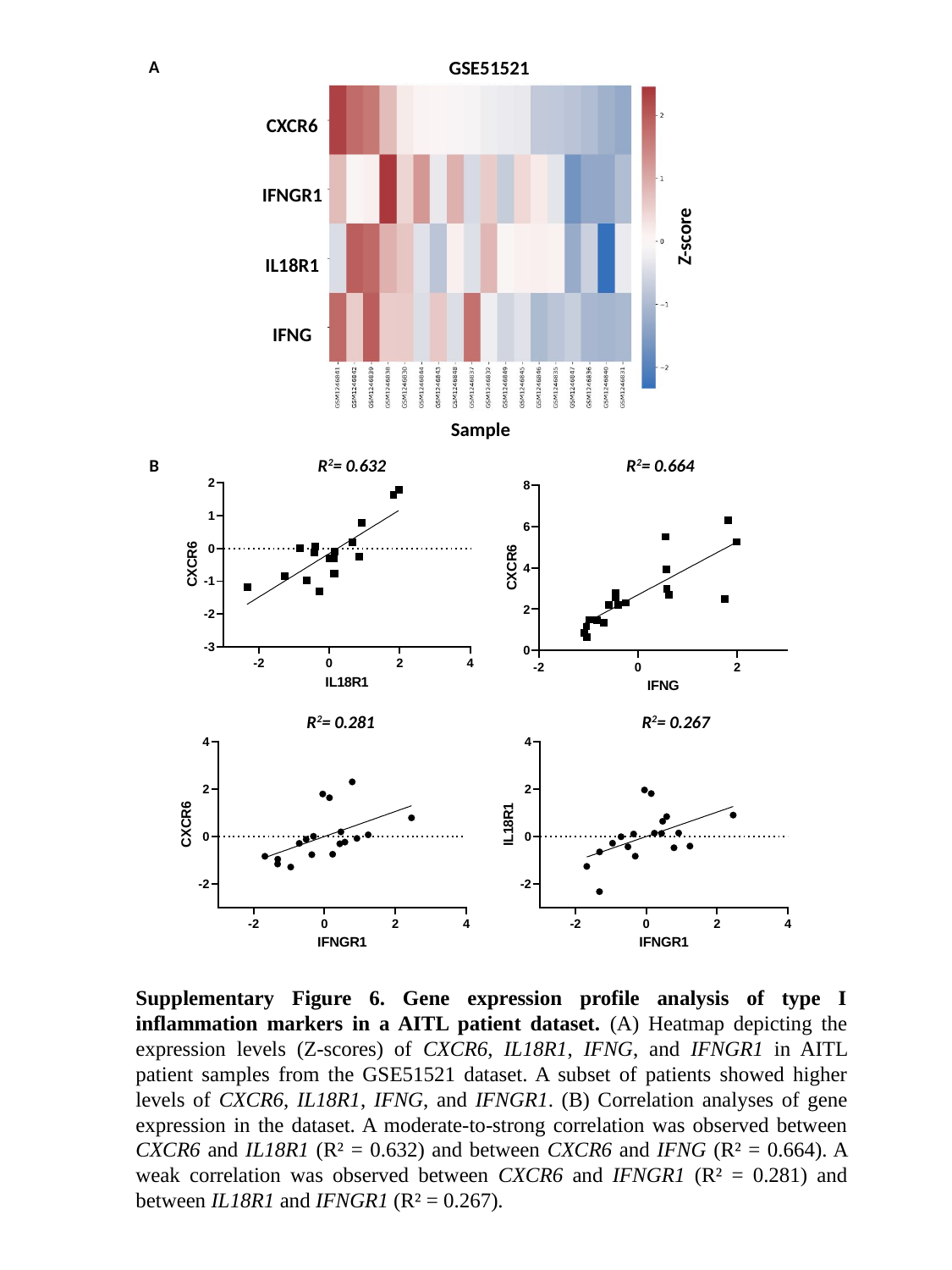

GSE51521
CXCR6
IFNGR1
IL18R1
IFNG
Z-score
Sample
A
B
R2= 0.632
R2= 0.664
R2= 0.267
R2= 0.281
Supplementary Figure 6. Gene expression profile analysis of type I inflammation markers in a AITL patient dataset. (A) Heatmap depicting the expression levels (Z-scores) of CXCR6, IL18R1, IFNG, and IFNGR1 in AITL patient samples from the GSE51521 dataset. A subset of patients showed higher levels of CXCR6, IL18R1, IFNG, and IFNGR1. (B) Correlation analyses of gene expression in the dataset. A moderate-to-strong correlation was observed between CXCR6 and IL18R1 (R² = 0.632) and between CXCR6 and IFNG (R² = 0.664). A weak correlation was observed between CXCR6 and IFNGR1 (R² = 0.281) and between IL18R1 and IFNGR1 (R² = 0.267).
